# Integration of Antioxidant Activity Assays Data of Stevia Leaf Extracts: A Systematic Review and Meta-Analysis

**DOI:** 10.3390/antiox13060692

**Published:** 2024-06-04

**Authors:** Maria Papaefthimiou, Panagiota I. Kontou, Pantelis G. Bagos, Georgia G. Braliou

**Affiliations:** 1 Department of Computer Science and Biomedical Informatics, University of Thessaly, 35131 Lamia, Greece; mpapaefthymiou@uth.gr (M.P.); pbagos@compgen.org (P.G.B.); 2 Department of Mathematics, University of Thessaly, 35131 Lamia, Greece; pkontou@uth.gr

**Keywords:** stevia, antioxidant assays, meta-analysis, meta-regression, polyphenols, flavonoids, oxidation status

## Abstract

*Stevia rebaudiana* Bertoni, a no-calorie natural sweetener, contains a plethora of polyphenols that exert antioxidant properties with potential medicinal significance. Due to the variety of functional groups, polyphenols exhibit varying solubility depending on the nature of the extraction solvents (water, organic, or their mixtures, defined further on as hydroalcoholic extracts). In the present study, we performed a systematic review, following PRISMA guidelines, and meta-analysis, synthesizing all available data from 45 articles encompassing 250 different studies. Our results showed that the total phenolic content (TPC) of hydroalcoholic and aqueous extracts presents higher values (64.77 and 63.73 mg GAE/g) compared to organic extracts (33.39). Total flavonoid content (TFC) was also higher in aqueous and hydroalcoholic extracts; meta-regression analysis revealed that outcomes in different measuring units (mg QE/g, mg CE/g, and mg RUE/g) do not present statistically significant differences and can be synthesized in meta-analysis. Using meta-regression analysis, we showed that outcomes from the chemical-based ABTS, FRAP, and ORAC antioxidant assays for the same extract type can be combined in meta-analysis because they do not differ statistically significantly. Meta-analysis of ABTS, FRAP, and ORAC assays outcomes revealed that the antioxidant activity profile of various extract types follows that of their phenolic and flavonoid content. Using regression meta-analysis, we also presented that outcomes from SOD, CAT, and POX enzymatic antioxidant assays are independent of the assay type (*p*-value = 0.905) and can be combined. Our study constitutes the first effort to quantitatively and statistically synthesize the research results of individual studies using all methods measuring the antioxidant activity of stevia leaf extracts. Our results, in light of evidence-based practice, uncover the need for a broadly accepted, unified, methodological strategy to perform antioxidant tests, and offer documentation that the use of ethanol:water 1:1 mixtures or pure water can more efficiently extract stevia antioxidant compounds.

## 1. Introduction

*Stevia rebaudiana* Bertoni is a perennial shrub well known for its use as a no-calorie natural sweetener [1]. In addition, whole stevia leaf preparations have been reported to contain a full bunch of chemical compounds classifying it in plant species such as *Ginko biloba*, *aloe vera*, *glycyrrhizin*, *ajwain*, *Withania somnifera*, *Solanum lycopersicum*, *Vitis vinifera*, *Malus pumila*, etc. that hold globally medicinal significance since they constitute a substantial source of natural antioxidant compounds [2,3,4,5,6,7,8,9]. Antioxidant compounds play a crucial role in maintaining health by combating oxidative stress, which is induced by the production of reactive oxygen species (ROS), endogenously or exogenously. Elevated systemic ROS levels have the potential to harm living organisms. Endogenous sources of ROS include metabolism, mitochondrial reactions, and inflammatory responses. Exogenous sources encompass radiation, air pollution, environmental carcinogens, toxins, alcohol, tobacco smoke, synthetic solvents, drugs, and dietary sources [10]. Under normal physiological conditions, there is a balance between ROS and endogenous antioxidant defense mechanisms. However, disturbances in this balance can lead to oxidative stress, resulting in cellular damage [11,12]. Consequently, oxidative stress can cause numerous diseases, such as cancer, cardiovascular diseases, atherosclerosis, diabetes, and inflammation disorders [13,14,15].

Cellular enzymatic antioxidant systems comprising the endogenous antioxidant defense system include superoxide dismutase (SOD), catalase (CAT), glutathione peroxidase (GPX), glutathione reductase (GR), and peroxidase (POX), whereas non-enzymatic systems comprise low-molecular-weight antioxidants such as phenols, flavonoids, terpenoids, catechins, carotenoids, and tannins [16,17]. Therefore, dietary antioxidants from plants, such as vitamin E, vitamin C, carotenoids, and polyphenols (flavonoids, phenolic acids), can enhance the activity of endogenous antioxidants [10,18], emerge as a promising tool to counteract the adverse consequences of oxidative stress.

Apart from the two main steviol diterpene glycosides, stevioside and rebaudioside A [19,20], stevia also contains other beneficial bioactive compounds, including phenols, flavonoids, phytosterols, chlorogenic acids, triterpenes, and crude fibers, with chlorogenic and caffeic acids being the most prominent phenolic secondary metabolites [21,22,23,24,25]. One of the most documented effects of *Stevia rebaudiana* leaf extracts is its antioxidant activity [1,26], due to its unique phenolic/flavonoid profile, which supports its use in the food industry as a strong natural antioxidant [27,28,29].

Polyphenols are diverse compounds bearing an aromatic ring and including phenolic acids, coumarins, flavonoids, stilbenes, and tannins [30,31]. The chemical nature of the individual groups, together with solvent polarity, imposes difficulties in developing a procedure suitable to extract all polyphenols from all plants [32]. A wide range of solvents, such as water, acetone, methanol, ethanol, or their mixtures with water, have been used for polyphenol extraction. Pure organic solvents exhibit a variety of properties, such as polarity, viscosity, surface tension, boiling point, volatility, density, and solubility in water, which influence their ability to interact with other substances [32,33]. Water is an efficient solvent in extracting polar molecules, while for less polar compounds, greater efficiency could be achieved by using organic or binary solvents [34]. The solubility of polyphenols in absolute organic solvents is low because of the high strength of hydrogen bonds between polyphenols and proteins, while solubility increases as water is added to organic solvents because the above hydrogen bonds become weaker in aqueous solutions [35,36]. Solvent mixtures of ethanol—an edible-safe alcohol—and water have been proven to be suitable systems for the extraction of plant phenolic compounds [34,37]. Therefore, scientifically synthesizing data on, and thereby proposing, an extraction method capable of extracting all phenolic components present in plants constitutes an absolute need and a great challenge.

Total phenolic content (TPC) and total flavonoid content (TFC) are commonly used as coarse indexes of the existence of antioxidants in plant extracts or plant-derived food products and therefore of their potential health value.

Total phenolic content (TPC) is determined using the Folin-Ciocalteu (F-C) assay, based on an electron-transfer reaction in which the antioxidant species under investigation acts as the electron donor, and the F−C reagent acts as the oxidant [38]. TPC is calculated using a standard curve, and the results are presented in the equivalents of reference phenols and gallic acid (GA), since plant extracts do not have a defined molar mass. Thus, TPC values are expressed as mg of GA equivalent per gram of dry sample (mg GAE/g).

**Figure 1:**
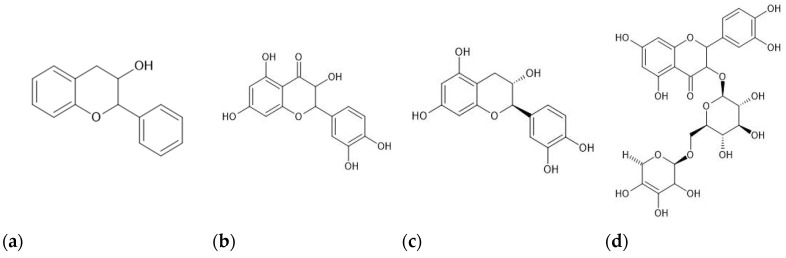
Chemical structure of (**a**) flavan-3-ol, (**b**) quercetin, (**c**) catechin, and (**d**) rutin. The chemical structures were created using ChemSketch Freeware version 2023.2.1 software.

Total flavonoid content (TFC) estimations are based on the formation of aluminum-flavonoid complexes [39]. TFC is calculated using a standard curve as milligram of the equivalent flavonoid compounds, namely, quercetin, catechin, or rutin, commonly found in plants. These compounds share a similar chemical structure, based on the structure of the flavan-3-ol as shown in Figure 1 (https://pubchem.ncbi.nlm.nih.gov/, accessed on 20 April 2024), and hence possess similar properties [40]. The results of TFC are expressed as milligrams of quercetin, catechin, and rutin equivalents per gram of dry sample [(mg QE/g), (mg CE/g), and (mg RUE/g), respectively] [39,41,42].

There are several in vitro assays used to measure the antioxidant activity of plant extracts, namely, the DPPH (2,2-Diphenyl-1-picrylhydrazyl) assay, the ABTS [2,2′–azinobis-(3-ethyl-benzothiazoline-6-sulphonicacid)] assay, the FRAP (ferric reducing antioxidant power) assay, and the ORAC (oxygen radical absorbance capacity) assay [2,43].

The DPPH assay is one of the most extensively used antioxidant assays for plant samples. DPPH is a stable free radical that reacts with compounds that can donate a hydrogen atom. The antioxidant activity (radical scavenging activity) [44] is expressed as the % inhibition (%I) of the sample compared to the control. Antioxidant activity, as calculated by the DPPH assay, is also expressed in mg or μmol of Trolox (6-hydroxy-2,5,7,8-tetramethylchroman-2-carboxylic acid) equivalent (TE) per gram of dry sample, (mg TE/g, μmol TE/g) [45], a water-soluble vitamin E analogue. In addition, DPPH values are also expressed as gallic acid, ascorbic acid, or rutin equivalents [46].

The ABTS radical scavenging assay is measured as an ABTS reduction in the presence of hydrogen-donating antioxidants. The FRAP assay is based on the capacity of antioxidants to reduce ferric ion Fe^3+^ to ferrous ion Fe^2+^ in a redox reaction. The most common measuring units are μmol TE/g and µmol Fe^2+^/g of dry sample [47]. Oxygen radical absorbance capacity (ORAC) is another assay for estimating antioxidant activity. Notably, it is the sole method that takes the free radical reaction to completion and uses an area under the curve (AUC) methodology for quantification, and antioxidant activity is expressed in μmol TE/g of dry sample [43].

Enzymes such as SOD (superoxide dismutase), CAT (catalase) and POX (peroxidase), are also commonly used to quantify the antioxidant activity of fresh food products [48]. The enzymatic activity of an enzyme is measured in units. One unit is defined as the quantity of the enzyme that catalyzes the conversion of one micromole of substrate per minute (μmol/min) under specified conditions of the assay method. The enzyme activity is expressed in units per gram of fresh tissue (U/g FW) or units per milligram of protein (U/mg protein) [49]. It is worth noting that enzymatic antioxidant assays are not as widely employed compared to chemical-based direct assays (TPC, TFC, DPPH, ABTS, FRAP, and ORAC). Enzymatic assays are predominantly employed in in vivo studies (in plants and animals) due to their applicability in the biological context of fresh tissue [29]. Measurement of malondialdehyde (MDA) content has also been used as a lipid peroxidation marker related to oxidative stress. During the process of lipid peroxidation, malondialdehyde (MDA) is formed by the decomposition of polyunsaturated fatty acids, which react with thiobarbituric acid. The thiobarbituric acid-reactive substances (TBARS) assay [50] is the most used method for both plant and animal samples for determining lipid peroxidation [51,52].

Accumulated data on the antioxidant activity of stevia leaf extracts exist in the literature, and their increasing rates, coming from a multitude of assays, show controversies among them [9,53,54,55]. Thus, it has become imperative to combine them in order to provide more accurate estimates, so that scientists can develop independent views regarding the antioxidant value of a stevia product. Meta-analysis constitutes a particular type of research in which a set of original studies’ research results are quantitatively synthesized and the potential diversity across them is explored using specific statistical methods [56,57,58]. Meta-analysis is a systematic, transparent, reproducible, and amended to guidelines for search strategy, screening, data extraction, research synthesis, inclusion criteria, quality assessment, statistical methods [(e.g., PRISMA database (preferred reporting items for systematic reviews and meta-analyses) https://www.prisma-statement.org/)], methodology to address well-defined scientific questions that can help establish evidence-based scientific practice to resolve research outcomes that seem—or are—contradictory [56,59]. It is a well-accepted and acknowledged methodology implemented thus far in psychology [60], biology [61,62,63], medicine [58,64], genetic epidemiology [65,66], pharmacogenetics, pharmacogenomics [67,68], diagnostics [69,70], and other scientific fields [71]. Meta-analysis falls in the category of evidence-based scientific practice (metascience), which uses scientific methodology to study science in an effort to identify methodological flaws, inefficiencies, and poor practices in research across numerous scientific fields and provide findings that can become guidelines for reproducibility, particularly in medicine. Metascience has been developed as a response to the “replication crisis” as part of a growing awareness of the problem [72,73].

The objective of the present study is to statistically synthesize existing data in the literature, combine them on the same scale to provide readily interpretable and scientifically meaningful effect sizes, and conduct comparisons of all available antioxidant activity assays on stevia leaf extracts. We set out to apply the meta-analysis methodology to these sets of data, encountering their specific features. We could perform statistically based (and not obligatory chemically based) comparisons of different assays, each employing various measuring units, and test sampling distribution. Thus, broad generalizations from a large number of studies’ outcomes can be attained, and a more comprehensive picture compared to each individual study can be reached. To this end, considering the mechanistic broadness of the diverse methods, the plethora of measuring units, and the need to compare results from different assays [53,54,55] that determine the antioxidant activity of plants (including stevia), an investigation was undertaken to explore the feasibility of combining specific methods or measurement units.

## 2. Materials and Methods

### 2.1. Literature Search Strategy and Eligibility Criteria

A comprehensive literature search was conducted in the PubMed database (https://pubmed.ncbi.nlm.nih.gov/) to retrieve all potential research articles exploring the antioxidant effects of stevia by means of in vitro assays. The search was lastly performed on 28 December 2023, using as the keywords “Stevia” and “antioxidant”, following the preferred reporting items for systematic reviews and meta-analyses (PRISMA) (http://www.prisma-statement.org/) [74], guidelines along with the advice for best practices [75]. To eliminate the implications of “gray literature bias”, articles in various languages were taken into consideration [76]. Eligible criteria for inclusion in the meta-analysis were: (a) assessment of the phenolic or flavonoid content of stevia leaf extracts, (b) (bio)chemical based in vitro antioxidant assays in stevia leaf extracts, (c) enzymatic-based assays that assess the antioxidant activity of fresh stevia leaves, and (d) antioxidant assays for stevia isolated glycosides. Articles with in vivo studies conducted on humans or animals, observational or intervention studies, and reviews were excluded. Studies that did not provide sufficient information or data necessary for the analysis were also excluded to ensure the reliability and validity of the results [77]. The systematic review and meta-analysis are registered in the OSF (Open Science Framework) (https://osf.io/) under the https://doi.org/10.17605/OSF.IO/5U7MQ.

### 2.2. Data Extraction

Initially, titles and abstracts of the articles were screened, and relevant articles were further examined in accordance with the inclusion and exclusion criteria. Search results were assessed by two separate researchers (M.P. and P.Ι.Κ.), and any discrepancies were discussed with G.G.B. and P.G.B. and agreed upon by consensus. The study enrolled articles employing all methods for in vitro assessing the phenolic and flavonoid content and profile, antioxidant capacity, oxidative stress parameters, and antioxidant enzymatic activity of stevia leaf samples. Data extraction was performed on a predetermined Microsoft Excel^®^ sheet. From each study, the following information was recorded: first author’s last name, publication year, country, type of assay, measuring units, type of stevia leaf sample, other specific characteristics, number of experimental repetitions, outcomes (effect sizes) of each experiment, along with standard deviations (SD) or standard error (SEM). For studies reporting SD values, the number of replicates was used to calculate SEM by the following type: $SEM=\frac{SD}{\sqrt{n}}$. For studies not reporting SD or SEM, a SEM value was imputed, which was equal to the biggest value reported for a certain category of compound [78].

### 2.3. Statistical Analysis

The random effects meta-analysis [79] was conducted using TPC, TFC, compound concentration, and antioxidant activity of each study as the effect size and its standard error. The between-study heterogeneity was evaluated using the chi-square-based Cochran’s Q statistic and the consistency index (I^2^). The presence of publication bias was evaluated with the use of Begg’s rank correlation test [80] and Egger’s regression test [81]. Subgroup analysis was performed according to the type of extract and the antioxidant method. Moreover, to determine whether different methods for antioxidant activity and measuring units can be combined in a meta-analysis, meta-regression analysis [82,83] was also used. Meta-analysis and meta-regression analysis were performed using the statistical software Stata version 13.1 [84]. The statistical significance was set at *p*-value less than or equal to 0.05.

## 3. Results

### 3.1. Studies Selection and Characteristics

**Figure 2:**
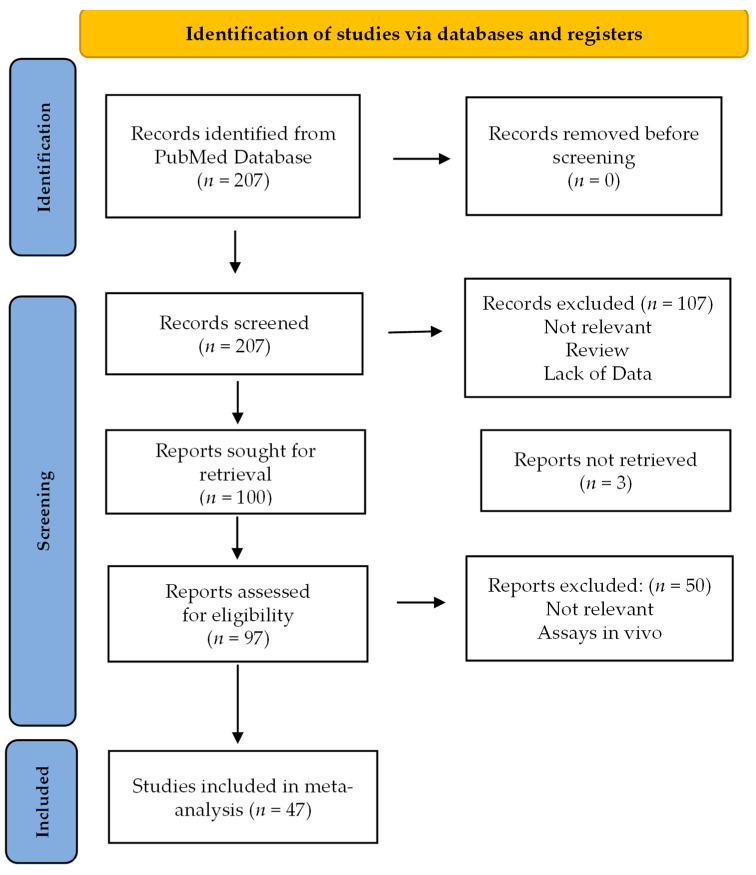
Flow diagram of systematic review to retrieve the selected studies for meta-analysis in accordance with the PRISMA statement.

A thorough literature search, in compliance with PRISMA Guidelines (http://www.prisma-statement.org/) for antioxidant activity of stevia resulted in 207 articles. After screening titles and abstracts and scrutinizing references lists, we identified 47 eligible articles comprising a total of 416 distinct studies [9,28,85,86,87,88,89,90,91,92,93,94,95,96,97,98,99,100,101,102,103,104,105,106,107,108,109,110,111,112,113,114,115,116,117,118,119,120,121,122,123,124,125,126,127,128,129] (Figure 2).

**Figure 3:**
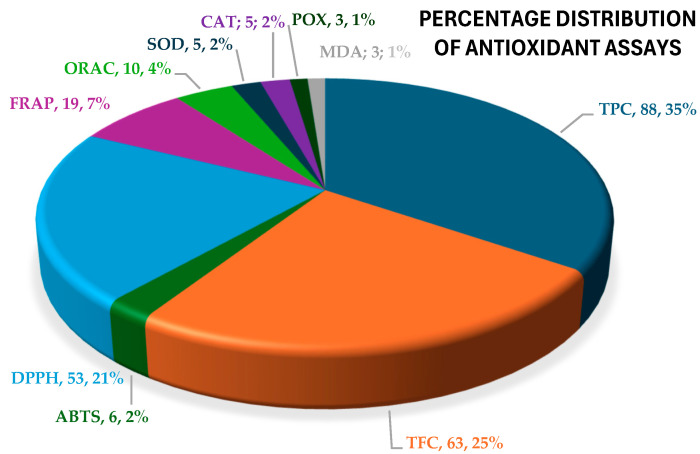
Pie diagram of absolute number and percentage distribution of studies on stevia antioxidant activity included in the present meta-analysis.

In all selected studies, antioxidant activity was assessed for stevia leaf extracts, including fractions enriched in phenols, flavonoids, and glycosides. The extractions were performed with the use of water (aqueous extracts), various organic solvents (organic extracts) and water:alcohol mixtures, hence called hydroalcoholic extracts [91,114]. The mixture of solvents in hydroalcoholic extracts varied in the type and the % content of alcohol (methanol from 80% to 85% and ethanol from 12% to 80%). Organic solvents used included methanol, ethanol, acetone, acetonitrile, chloroform–methanol, ethyl acetate, and DMSO. TPC was investigated in 88 studies and TFC in 63 studies, while phenolic/flavonoid profile was investigated in 166 studies. Antioxidant activity was measured with the use of chemical-based assays in 88 studies [ABTS (6 studies), DPPH (53 studies), FRAP (19 studies) and ORAC (10 studies)], while with enzymatic assays in 13 studies [SOD (5 studies), CAT (5 studies), and POX (3 studies)] and with MDA in 3 studies (Figure 3).

Since polyphenols and flavonoids exert powerful antioxidant action [10,16,18], we documented data on TPC (88 studies) and TFC (63 studies) values. TPC values were expressed in gallic acid equivalents (mg GAE/g), and tannic acid equivalents (mg TAE/g—one study) of dry leaf sample (Table 1). TFC was assessed across studies with the use of different units, including milligrams of quercetin equivalents per gram of dry leaf sample (mg QE/g) (23 studies), milligrams of catechin equivalents (mg CE/g) (24 studies), and milligrams of rutin equivalents (mg RUE/g) (14 studies) of dry leaf sample. Concentrations of phenolic/flavonoid individual compounds were given in mg/g (4 studies) and in mg of chlorogenic acid equivalents (mg CGAE)/g of dry leaf (40 studies). The ABTS assay was measured in micromoles of Trolox equivalents per gram of dry leaf (μmol TE/g) (five studies); one study reported percentage inhibition (%I) measured with the ABTS assay. The radical scavenging activity (RSA) evaluated by DPPH assay was expressed as percentage inhibition (%I) (30 studies). Additionally, DPPH assay values were expressed in milligrams of Trolox equivalents (mg TE/g) (16 studies), Ascorbic Acid (mg AAE/g) (5 studies), and Rutin (mg RUE/g) (2 studies) per gram of dry leaf sample. Trolox and Fe^2+^ were used as standard reference compounds in the FRAP assay, hence, the values were expressed in Trolox equivalents (μmol ΤΕ/g of dry leaf sample) (12 studies) and Fe^2+^ equivalents (μmol Fe^2+^/g of dry leaf sample) (7 studies). Finally, the ORAC assay values were expressed in Trolox equivalents (µmol TE/g of dry leaf sample) (10 studies).

As already mentioned, enzymatic antioxidant assays are not widely employed for direct assays on fresh tissues. So, only five studies reported data from measuring SOD activity, five studies from CAT, and only three studies from POX activity. POX enzyme activity was expressed only in U(units)/g of fresh weight tissue (FW), while SOD and CAT activities were expressed in U/g FW (three studies). Finally, MDA content results (three studies) were expressed in mM/g FW.

Some articles included several specific characteristics (Table 1), such as the year of the experiments [122], harvest time [110], stevia varieties, genotypes, cultivation locations, and samples sourced from various companies [89,91,95,109,116]. Furthermore, factors such as extraction methods, solvents, temperatures, extraction times [86,99,105,130], and the manner of sample dryness were also considered [92,117,118].

### 3.2. Meta-Analysis of Total Phenolic, Total Flavonoid Content, and Their Profiles of Stevia Leaf Extracts

**Figure 4:**
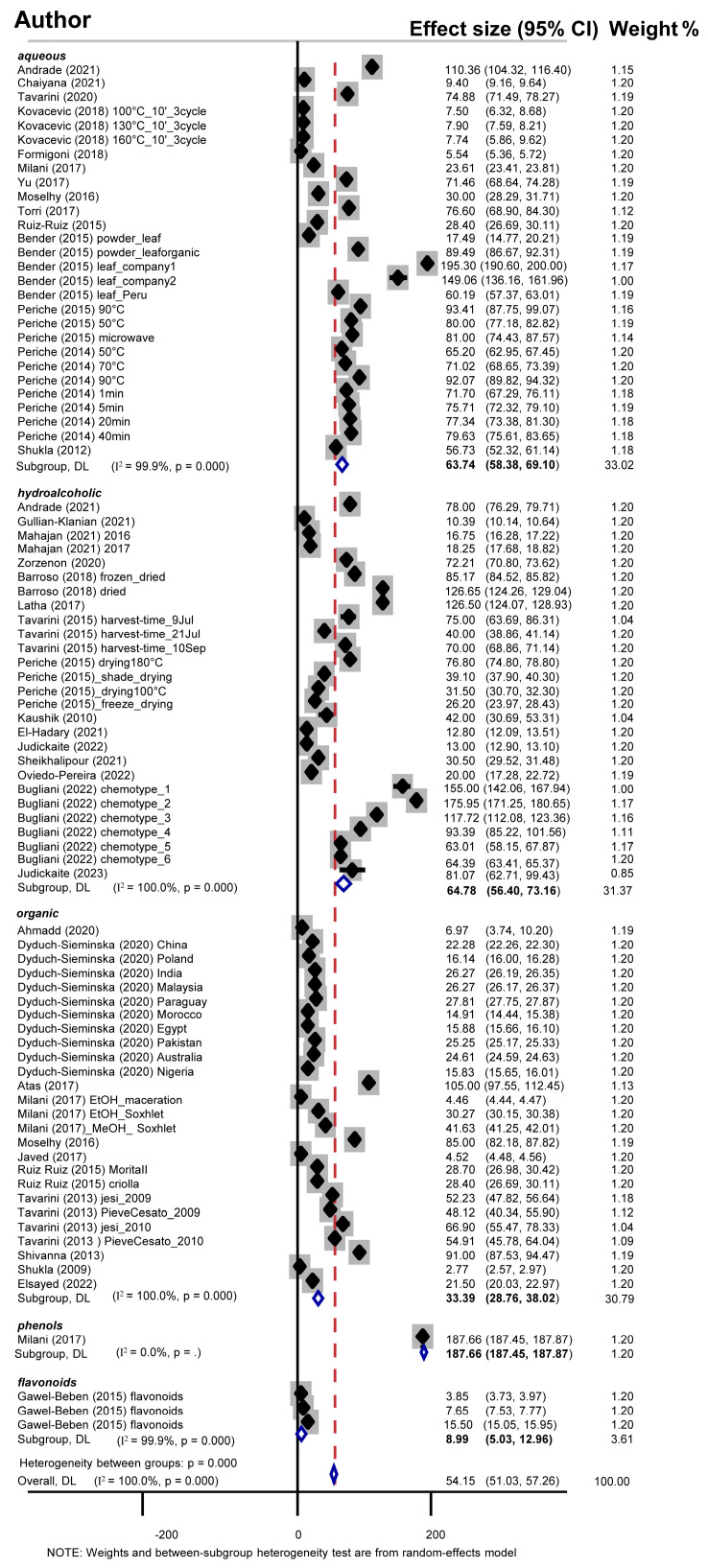
Forest plot of the effect size and 95% confidence interval of total phenolic content. Blue open rhombuses depict overall effect sizes.

Since plant antioxidants belong to the class of polyphenols, including flavonoids, it was important to primarily investigate the TPC of stevia leaf extracts. Considering that polyphenols bear groups with different polarities, they present varying solubility in various common solvents. Thus, meta-analysis was performed separately for aqueous (28 studies), hydroalcoholic (27 studies), and organic (26 studies) extracts (Figure 4).

As shown in Table 2, meta-analysis revealed that hydroalcoholic extracts demonstrated TPC values of 64.77 mg GAE/g dry leaf, while the aqueous ones exhibited a slightly lower content of 63.73 mg GAE/g. Conversely, extraction with pure organic solvents yielded a remarkably reduced TP content of 33.39 mg GAE/g. We also encountered studies investigating TPC in polyphenol-enriched and flavonoid-enriched fractions. Meta-analysis showed a notably high TPC of 187.66 mg GAE/g and a very low (8.99 mg GAE/g) in polyphenol- and flavonoid-enriched extracts, respectively. However, these results should be considered with caution since they are based on very limited available data (Table 2).

Considering solvent toxicity, since methanol is recognized for its higher toxicity compared to ethanol, stratification analysis was performed according to the organic constituents of the hydroalcoholic solvent mixtures. The use of ethanol (20 studies) resulted in a higher TPC of 71.26 mg GAE/g compared to the use of methanol (6 studies), with a value of 47.93 mg GAE/g of dry leaf sample. Further stratification according to the alcohol-to-water ratio of hydroalcoholic extracts demonstrated that the most efficient extraction, in terms of TPC, was attained with 50% ethanol reaching 113.60 mg GAE/g (seven studies), and with 80% methanol (five studies) reaching 51.41 mg GAE/g. Due to the limited number of studies, robust results could not be obtained for methanol 85%, ethanol 12%, ethanol 75%, and ethanol 80% solvent mixtures. When pure organic solvents were encountered, stratification analysis revealed a higher TPC of methanol extracts (30 studies), reaching 30.58 mg GAE/g, followed by acetone extracts (3 studies), with 25.12 mg GAE/g. Because of the limited available data on acetonitrile, chloroform–methanol, ethyl acetate, and DMSO extracts, conclusive results could not be reached for the last solvents (Table 2).

**Figure 5:**
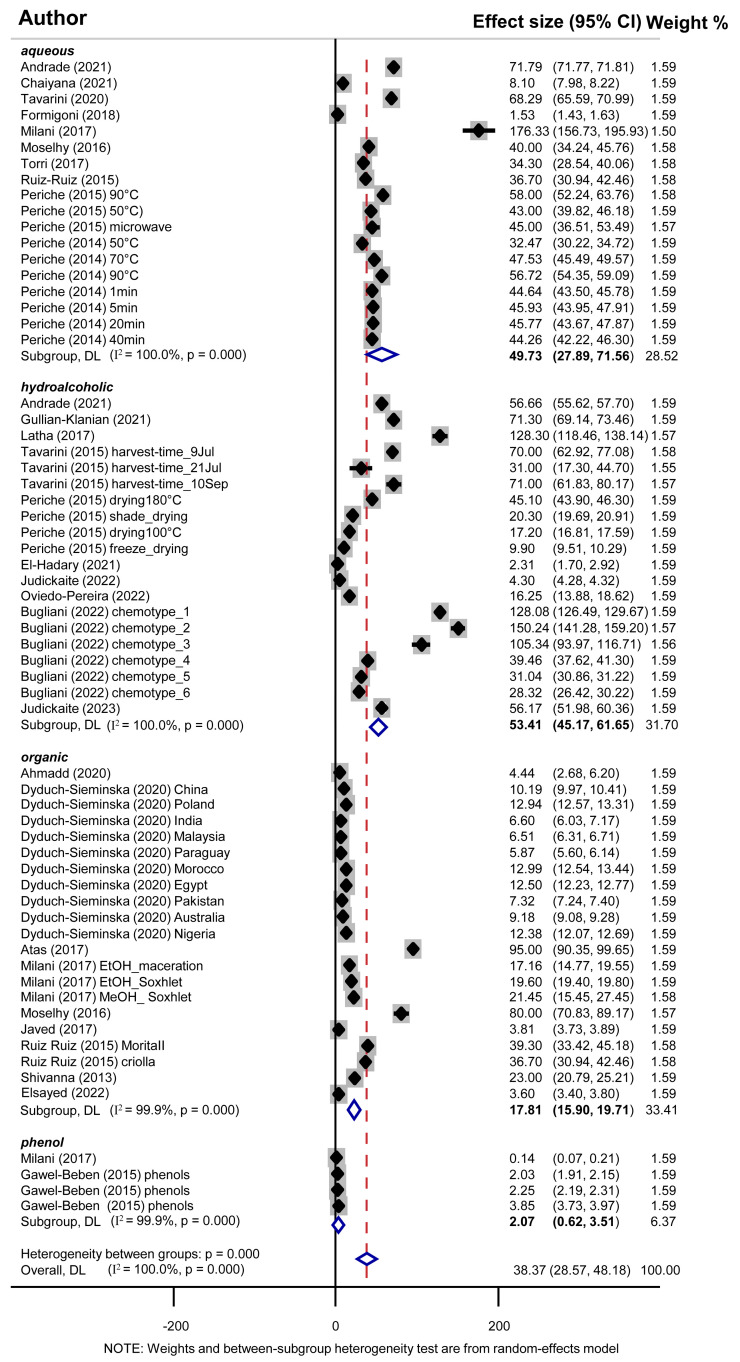
Forest plot of the effect size and 95% confidence interval of total flavonoid content. Blue open rhombuses depict overall effect sizes.

Subsequently, we initiated a thorough meta-analysis to assess the TFC of stevia leaf extracts as well as the influence of different solvents on TFC across studies. However, three different measuring units were used in the studies, that is, mg QE/g, mg CE/g, and mg RUE/g. Thus, we first employed a comprehensive meta-analysis for all units for each type of extract, and at the same time, we stratified for each unit [mg QE/g (25 studies), mg CE/g (24 studies), and mg RUE/g (14 studies)] (Figure 5 and Table 3). Similarly to the observations on TPC values, TFC was marginally higher in hydroalcoholic extracts (53.41 all units) compared to aqueous (49.72), and higher than organic ones (17.80). Stratification meta-analysis according to different measuring units demonstrated higher TFC values (54.90 mg QE/g) in aqueous extracts, moderate TFC in hydroalcoholic extracts (36.62 mg QE/g), while organic solvents showed the lowest content of 29.78 mg QE/g (Table 3). Additionally, TFC in the phenol fraction (4 studies) was notably low at 2.07 mg QE/g. Measurements for TFC in mg CE/g and mg RUE/g showed higher values in hydroalcoholic extracts compared to aqueous or organic ones (Table 3). Egger’s and Begg’s tests did not detect publication bias.

**Figure 6:**
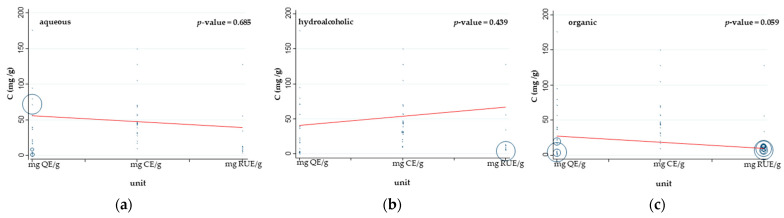
Meta-regression analysis for TFC outcomes (in mg/g of dry leaf sample) for all measuring units (mg QE/g, mg CE/g, and mg RUE/g) for (**a**) aqueous extracts, (**b**) hydroalcoholic extracts (solvent mixture of water and alcohol), and (**c**) organic extracts. The size of each circle is representative of the weight of each study.

We next wondered whether variance in measuring units could affect the statistical synthesis of studies’ outcomes and confer between-study heterogeneity in the meta-analytical synthesis. To test this, a meta-regression analysis for TFC data across all measuring units was conducted separately for each type of extract. No statistically significant differences were found among various measuring units for any of the three types of extracts since *p*-values were 0.685 for aqueous, 0.439 for hydroalcoholic, and 0.059 for organic extracts (Figure 6). As a result, the TFC values of stevia leaf extracts are independent of these three measuring units and can be treated as coming from the same source, allowing their combination in subsequent meta-analyses.

Thus, our initial comprehensive meta-analysis encompassing data on all units (Figure 5) is valid, and hydroalcoholic extracts indeed show a higher TFC value of 53.41 mg of all equivalent compounds/g dry leaf sample compared to aqueous and organic extracts.

Subsequent stratification meta-analysis for various alcohol:water ratios for hydroalcoholic extracts, integrating all units, revealed that the highest extraction efficiency of flavonoid compounds can be attained with 50% ethanol (TFC 87.05 mg/g), followed by 70% ethanol (TFC 46.47 mg/g). Due to the limited number of studies, robust results for other methanol:water and ethanol:water ratios could not be obtained. Furthermore, stratification according to the pure organic solvent revealed the highest TFC with the use of ethanol at 18.68 mg/g in two studies compared to methanol. However, this difference is marginal and should be considered with caution (Table 3).

Given that the phenolic/flavonoid profile of each plant extract is unique [24], we subsequently wanted to investigate the content of stevia leaf extracts in individual compounds, with the aim to correlate TPC, TFC, and specific metabolites with the antioxidant activity of various extracts. From the initial 47 articles, further data were extracted (Supplementary Table S1), encountering additional studies that provided information on individual polyphenols [9,129]. In total, eight articles (encompassing 166 studies) provided concentration information for the phenolic/flavonoid profile. However, meta-analyses were performed only if at least three studies existed providing concentration data (in transformable units) for the same compound, measured in the same type of extract; thus, we enrolled data from five articles [88,89,96,120,129] containing 54 studies. As shown in Table 4, the most prominent chlorogenic acid in aqueous stevia leaf extracts is 3,5-dicaffeoylquinic acid (3,5-diCQA) with 50.53 mg of chlorogenic acid equivalents per g of dry leaf sample, followed by 4,5-diCQA and 4-CQA (18.69 and 11.85 mg CGAE/g dry leaf, respectively). Importantly, the contents of aqueous and hydroalcoholic extracts in 3-CQA are comparable, a finding that is in accordance with the correlation of TPC and TFC in aqueous and hydroalcoholic extracts (Table 2 and Table 3). Of note is the finding that the concentration of 5-CQA is much higher in organic (chloroform–methanol) extracts compared to aqueous extracts (23.39 compared to 1.65), a finding also mentioned by the authors of the original studies [9,129], that warrants further investigation.

### 3.3. Meta-Analysis of Antioxidant Assays: DPPH, FRAP, ABTS, and ORAC

After having seen the concordance between meta-analyses results for TPC and TFC of various types of stevia leaf extracts, which highlighted the high content of phenol and flavonoids in hydroalcoholic extracts, we opted to further explore the antioxidant activity of the above extracts as estimated with DPPH, FRAP, ABTS, and ORAC methods.

#### 3.3.1. Meta-Analysis of DPPH Assay Data

The DPPH assay estimates the antioxidant activity of a sample by measuring the potential of substances to serve as hydrogen providers or free-radical scavengers. DPPH results are expressed in two different ways: (a) % inhibition (radical scavenging activity) and (b) mg of equivalent compound g of dry leaf sample. A meta-analysis of DPPH assay outcomes, measured as % inhibition, included four studies for hydroalcoholic, four for aqueous, and 21 for organic extracts. As shown in Table 5, the effect sizes were 72.31%, 73.09%, and 55.84%, respectively, showing that the use of water or ethanol-water mixtures extract comparable antioxidant activity to stevia leaf extracts. These results are in agreement with TPC and TFC meta-analyses outcomes, showing similar contents between aqueous and hydroalcoholic extracts.

Additionally, DPPH assay results were expressed in milligrams of equivalent compounds per gram of dry sample. Consequently, a meta-analysis of the results from the DPPH assay was conducted cumulatively for all equivalent compounds and for each unit as well. Before incorporating all measuring units in the meta-analysis, we set out to test whether results obtained comprehensively from all measuring units could be synthesized in a statistically significant manner. Thus, a meta-regression analysis for DPPH data for hydroalcoholic extracts across all measuring units (mg TE/g, mg AAE/g, and mg RUE/g) was conducted and revealed non-statistically significant differences (*p* = 0.144) (Figure S1). Meta-regression analyses for aqueous and organic extracts were pointless since these values were expressed in a single measuring unit. As a result, DPPH datasets for hydroalcoholic extracts across these three measuring units can be treated as coming from the same source and can be quantitatively synthesized in subsequent meta-analyses.

Meta-analysis combining all units revealed that the antioxidant activity of aqueous extracts was higher (92.80 mg/g) than hydroalcoholic extracts, which exhibited 46.17 mg of all reference-equivalent compounds/g of dry leaf (Table 5). Stratification according to measuring units revealed that, when measured in mg ΤΕ/g, aqueous extraction exhibited a higher antioxidant activity of 92.80 mg ΤΕ/g compared to hydroalcoholic extracts, which reached 67.96 mg ΤΕ/g. Meta-analysis for DPPH values expressed in mg RUE/g could be performed only for hydroalcoholic extracts due to the lack of data for both aqueous and organic extracts (Table 5). Publication bias (Egger’s and Begg’s tests) was not detected.

#### 3.3.2. Meta-Analysis of FRAP, ABTS, and ORAC Assays Data

The ferric reducing antioxidant power (FRAP) assay was assessed across studies using two different measuring units, namely, micromoles of Fe^+2^ per gram (µmol Fe^+2^/g) and micromoles of Trolox per gram (µmol TE/g) of dry leaf sample. Comparably to the analysis performed for DPPH assay data, we also wished to perform meta-analysis for all FRAP assay data and separately for each measuring unit as well. For this, we first performed a meta-regression analysis for FRAP data across all measuring units. Since the data given in both measuring units were available only for aqueous extracts, meta-regression was performed only for them and revealed no statistically significant difference since the *p*-value = 0.649. Thus, FRAP datasets across these two measuring units can be treated alike, allowing their combination in subsequent meta-analyses.

Meta-analysis was then performed using the combined data from the various measuring units (Table 6). The analysis showed that the results for hydroalcoholic solvent from 10 studies (measured in µmol TE/g) showed a higher antioxidant activity of 855.53 µmol/g, while aqueous solvent in 3 studies demonstrated a lower value at 320.01 µmol/g. Stratification meta-analysis according to the ethanol:water ratio of hydroalcoholic mixtures revealed that extracts obtained with a 1:1 solvent ratio exhibited the highest antioxidant activity (Table 6), a finding that is in agreement with the TPC and TFC results showing the best polyphenol and flavonoid extraction efficiency with 1:1 ethanol:water mixtures. Extraction with an organic solvent showed the lowest antioxidant activity at 248.40 µmol Fe^+2^/g across six studies.

The ABTS assay results are given in μmol of Trolox equivalents (μmol TE/g) of a dry leaf sample. Meta-analysis of ABTS results demonstrated higher antioxidant activity of aqueous extracts (680.00 μmol TE/g) compared to hydroalcoholic extracts (581.44 μmol TE/g) and organic ones (313.64 μmol TE/g) (Table 6). However, these results should be interpreted with caution since they are derived from a limited number of studies that are available in the literature.

The ORAC assay results were also given only in μmol of Trolox equivalents (μmol TE/g) of dry leaf sample. Meta-analysis of results from the ORAC assay showed that aqueous extracts have higher antioxidant activity compared to hydroalcoholic extracts, that is, 879.28 versus 454.90 µmol TE/g of dry leaf, respectively (Table 6). Because the available outcomes from FRAP, ABTS, and ORAC assays are all expressed in µmol TE/g of dry leaf, we were prompted to investigate whether these results could be synthesized in a meta-analysis. Thus, a meta-regression analysis was performed for all assays’ data on aqueous and hydroalcoholic extracts and showed non-statistically significant differences with *p*-values = 0.148 and 0.489, respectively (Figure S2).

In fact, a meta-analysis performed with data from 12 studies on aqueous extracts and 14 studies on hydroalcoholic extracts resulted in 723.15 µmol TE/g (95%CI: 522.79, 923.52) and 720.48 µmol TE/g (95%CI: 645.30, 795.65), respectively (Table 6). Overall, extracts with water or alcohol-water mixtures present comparable antioxidant activities as assayed with FRAP, ABTS, and ORAC assays, which is in accordance with findings from TPC, TFC, and DPPH assays. However, more experiments with the use of the same organic and hydroalcoholic solvents performed with all three assays, and subsequent stratification according to the alcohol:water ratios are expected to uncover important aspects of the antioxidant activity of stevia leaf extracts and verify the equivalency of the methods.

### 3.4. Activities of Enzymatic Antioxidant Systems (SOD, CAT, POX) and MDA

The activities of the three antioxidant enzymes, namely, superoxide dismutase (SOD), catalase (CAT), and peroxidase (POX), were quantified in fresh stevia leaves across five, five, and three studies, respectively. Enzymatic activity was expressed in two different ways: (a) units per gram of the fresh leaf weight (U/g) and (b) U/mg protein of the fresh leaf sample. We initially performed meta-analysis (Table 7) and found 98.44 U/g for SOD, 41.49 U/g for CAT, and 113.60 U/g for POX enzymatic assays. Meta-analysis for antioxidant enzyme outcomes expressed in U/mg protein could not be performed due to the small number of studies (two for each assay). We next wondered whether we could statistically synthesize the results obtained from the three antioxidant enzymes. To achieve this, a meta-regression analysis was conducted on the data from SOD, CAT, and POX, all expressed in U/g. Meta-regression analysis revealed a non-statistically significant difference among them (*p*-value = 0.905) (Figure S3), and thus their results can be synthesized in subsequent meta-analyses. As shown in Table 6, meta-analysis with data from SOD, CAT, and POX assays from nine studies revealed enzymatic antioxidant activity of 114.42 U/g.

The malondialdehyde (MDA) assay assesses lipid peroxidation and functions as a marker related to oxidative stress. Although it is not an antioxidant enzyme-based assay, it is included in this section due to its applicability to fresh tissue samples. Meta-analysis of MDA data from three studies showed an MDA content of 1.95 mmol per gram of fresh leaf samples (mM/g) (Table 7).

## 4. Discussion

Stevia is a well-known no-calorie natural sweetener, yielding 8−13% steviol glycosides (SGs) from dried leaves that comprise 64 different SGs. However, a substantial 2%-5% of these dried leaves constitute the polyphenol portion, which contains more than 30 various stevia phenolic compounds [131]. In general, phenolic compounds primarily help plants survive infections and injuries by maintaining their oxidative stability. Thus, edible plants are assumed to convey this antioxidant health-beneficial potential to humans when consumed, classifying them as “functional foods”. Antioxidant activity is considered to be the potency of a compound to inhibit the oxidation of its substrate [132]. The Folin-Ciocalteu-based TPC assay is primarily used as an important indicator of plant health value in terms of antioxidants. However, due to the multifaceted nature of antioxidants that may act in different pathways, multiple other assays have been developed to precisely determine the antioxidant activity, using as references various antioxidant compounds [131]. For example, the chemical methods ABTS, DPPH, FRAP, or ORAC can measure the potency of an antioxidant to inhibit the oxidation of its substrate. They are all relatively simple, quick, and cheap. Although chemical-based methods belong to two categories [hydrogen atom transfer reaction (HAT) and single-electron transfer reaction (ET)-based assays], DPPH and ABTS combine the two mechanisms. In addition, ABTS, DPPH, and FRAP are used with skepticism because they measure the inhibition of radicals that do not exist in biological systems, and thus, they may have limited biological relevance [133]. On the contrary, the ORAC assay reports on the inhibition of the biologically relevant peroxyl radical by detecting the quenching capacity of different oxygen and reactive nitrogen species [134]. Although the use of assays of biological relevance is highly encouraged (to avoid possible over- or underestimations due to the absence of a biological system), non-biologically relevant methods can lead the research towards more appropriate quantification methods [131]. The estimation of food antioxidants is becoming a further complicated task if we consider the existence of additional in vivo systems used to estimate the antioxidant potency of fresh stevia leaves. In addition, it is important to note that several studies report contradictory results even on the same extracts tested with different assays, let alone when testing extracts from various solvents and solvent ratios.

The increasing accumulation of data on the antioxidant properties of stevia and other plant extracts reflects the need for the field to uniformly characterize their antioxidant parameters and adopt [53,55,131,135] standardized antioxidant testing. Although it is widely accepted that one methodology cannot be used to evaluate all nutritional and health aspects of a food, including antioxidant activity, the food industry, free market laboratories, and consumers (end users) require a very small number of indexes (preferably one) that can describe and compare the health value of plant products [53,54,55]. In such cases, meta-analysis can help to quantitatively summarize available data from various methodological approaches on a specific theme and propose certain methodologies with more confidence that can be used as gold standards [135,136,137]. Due to its specific approach, meta-analysis can achieve generalizations of an effect that is dealt with in a large number of studies that present heterogeneity between them. Moreover, it can identify sources of heterogeneity in outcomes and, thus, shed light on the overall phenomenon and help examine factors that modify outcomes [56]. In this direction, the present meta-analysis constitutes the first attempt to quantitatively synthesize all publicly available data on the antioxidant activity of stevia leaf extracts and propose flows of laboratory methodologies that can be further investigated for their use as reference methods. This meta-analysis is not meant to comment on the advantages and disadvantages of each technique and uncover the most appropriate; rather, it is designed to conduct data synthesis and comparisons based on the type of sample and solvent mixtures used to prepare extracts in an effort to uncover factors that modify test results in order to minimize the present chaos in methodologies used to calculate plant antioxidant activity [55].

Given that TPC is a good, yet not the ultimate, indicator for antioxidant activity, we initially performed a meta-analysis with TPC data. Our findings suggest a comparable phenolic content in aqueous and hydroalcoholic (solvent mixtures of water and alcohol) stevia leaf extracts, which is significantly higher compared to extracts made with individual organic solvents. For the hydroalcoholic extracts, ethanol seems to hold higher extraction potency than methanol, while the alcohol-to-water ratio does affect the extraction efficiency, with 50% being the best combination to extract polyphenols. Similar results were obtained for TFC data in terms of the extracting solvent used. Meta-regression analysis showed that, despite the different reference compounds used, the exact measuring units do not critically affect TFC values. Again, 50% ethanol seems to more efficiently extract stevia flavonoids compared to any other solvent. Moreover, meta-analysis of data for individual phenolic compounds revealed a composite common profile of chlorogenic acids (esters of quinic or shikimic acid) in aqueous stevia leaf extracts, with 3,5-dicaffeoylquinic acid being the most abundant, followed by 4,5-dicaffeoylquinic and 4-Caffeoylquinic acids, which is in accordance with findings from Wanyo and coworkers [24] and Zhang et. al. [138] and as reviewed in [25,131].

In terms of free radical scavenging activity of the extracts, as measured with the DPPH method expressed in % RSA, aqueous and hydroalcoholic extracts conveyed similar activity and were higher than that of pure organic extracts. Meta-regression analysis, recruited to investigate if different expression units for the DPPH assay introduce variation in study outcomes, revealed that study outcomes did not depend on them and that they could be synthesized and compared. Meta-analysis of such data revealed that aqueous extracts possess higher antioxidant activity compared to hydroalcoholic and organic ones. Yet, given the shortage of data on certain extract types expressed in specific units, this conclusion awaits verification or rejection by the addition of further experimental outcomes, when performed. Intriguing is also the finding that a fraction of stevia extract containing only glycosides held remarkable antioxidant activity. Considering that SGs are complex molecules consisting of the sugar molecule and the aglycone, a stevia diterpene, it is not surprising that SGs can exert antioxidant activity. This is in accord with experiments showing that rat cardiac fibroblasts presented higher SOD and CAT activities when treated with stevia glycosides [139] and with results from a previous meta-analysis showing that treatment with stevia glycosides can restore the oxidative stress status of diseased rats that had received stevia glycosides [29].

Meta-analytical synthesis of FRAP assay data expressed in μmol TE/g revealed a higher antioxidant content in hydroalcoholic extracts compared to aqueous ones. More importantly, further stratification according to the solvent ratio of hydroalcoholic extracts revealed that a water:ethanol 1:1 mixture presented the best antioxidant activity. This is in accordance with findings from TPC and TFC assays showing that this water:ethanol 1:1 solvent ratio exhibits the highest extraction efficiency for polyphenols and flavonoids. In contrast to FRAP, meta-analyses of ABTS and ORAC assays data showed that aqueous extracts present higher antioxidant activity compared to hydroalcoholic or organic extracts. Although this discrepancy could lie on the chemical basis of the assays, that is, FRAP is ET-based, ORAC is HAT-based, ABTS is HAT-based, and ET-based [53,55], other reasons, such as exact extraction temperature, pH, maceration time, and other study design parameters, could have affected individual outcomes [131,135] that a meta-analysis cannot account for if information is not provided. The use of different alcohols and different alcohol-to-water ratios in hydroalcoholic extracts further imposes a confounding factor in the meta-analysis. The fact that meta-regression analysis of 12 studies on aqueous extracts (where only water was used as a solvent, which acts as a constant parameter in the meta-analysis contrast) revealed the non-statistically significant difference among the three assays results further supports the notion that FRAP ABTS and ORAC assay outcomes can be combined in a meta-analysis. Nonetheless, all our data point in the direction that water or water:ethanol 1:1 solvent mixture can extract most phenolic/flavonoid compounds. These extracts show the highest antioxidant activity, which can be attributed mainly to 3,5-dicaffeoylquinic acid and less to 4,5-dicaffeoylquinic and 4-Caffeoylquinic acids.

Enzymes as oxidative stress biomarkers have been extensively used to describe the oxidant status of a living cell [140]. In contrast to the previous chemical assays, the enzymatic SOD, CAT, and POX assays are performed with fresh stevia leaf samples. Enzymatic assays to assess the antioxidant potency of a plant product have been used since 1976 [141], yet, currently, they are preferentially used to describe the oxidant status of animals under various intervention schemes, such as stevia consumption [142]. Herein, a meta-analysis of studies performed with each assay reported comparable activities, while a meta-regression analysis verified that assay results do not depend on the type of these three enzymatic assays. This finding is of high importance since it allows us to pool data from similar but different sources and leads to increased power in the analysis. Moreover, this result is in line with our previous findings [29], showing that standardized mean difference results from SOD, CAT, and GPx assays performed in rats of various stevia intervention groups can be combined.

Our meta-analysis is subjected to certain limitations that constitute some of the challenges we had to face herein. As mentioned above, the variety of extract types (aqueous, hydroalcoholic, and organic) that was further increased by the plethora of water-to-organic ratios introduced heterogeneity to our analysis, which we encountered by stratifying our analysis. However, other parameters concerning the drying procedure of the fresh leaves, the particle size of the dried leaves, the extraction method, including maceration time, pH, temperature, pressure, solvent polarity during the preparation of extracts, or even the maintenance conditions of the extracts (influencing the stability of each phenolic compound [131,143]), could have added confusion to our meta-analysis. The antioxidant capacity of a stevia extract is also influenced by the culture conditions of the plant, including salinity, fertilizer dose, H_2_O_2_ stress, light, and soil pH [131,144]. Information on these parameters was not given in the included studies. Moreover, if individual profiles of the phenolic and flavonoid compounds were given along with outcomes from the same assays, we could have performed a head-to-head meta-analysis (comparing the antioxidant activities of the same phenolic or flavonoid compounds). To summarize, important metadata was lacking in all studies, and thus intriguing and informative tests could not be performed. Nevertheless, all meta-analyses were performed with caution, taking into consideration all possible parameters available. A high degree of heterogeneity was expected, and indeed it was observed in most of our analyses (Figure 4 and Figure 5), which can be attributed to flaws in individual study designs. Yet, in the present meta-analysis, we used the random-effects model, which assumes that there are both sampling variance and other variances inherent to the biological question investigated each time [145], including variance in extractability due to the varying polarity of the solvents. In addition, despite the intensive effort of the authors to systematically include all existing studies (conference proceedings, theses, published in all languages), we could not exclude the existence of “gray literature bias”. 

Finally, the current systematic review has uncovered the lack of widely agreed-upon and standardized protocols for evaluating the antioxidant activity of stevia leaf extracts, starting with sample collection, extract preparation, the performance of tests, and the ways that this performance is validated (in various measuring units). The present meta-analysis, by recruiting formal research synthesis methodology, has come to the point to propose that the use of water or water:ethanol 1:1 solvent mixture can almost equally efficiently extract stevia phenolic compounds with the highest antioxidant activity. Our findings support the global, oncoming change in scientific thinking, that is, an individual primary study may be seen in the context of accumulating evidence rather than presenting a conclusive answer to a scientific question. Thus, only if uniform settings are imposed could accurate comparisons of protocols be performed in order to get a more general and complete picture of stevia antioxidant activity, useful for the scientific community, the food industry, and end consumers.

## 5. Conclusions

Increasing research on artificial sweeteners [146] has emerged as a global awareness and effort to consume food products that support a healthier lifestyle [147]. In addition, considerations on the environmental impact of culture and the production of natural sweeteners are lined with sustainable practices and the circular economy of agriculture productions so that culture by-products can be exploited in medicine, pharmacy, and the food industry [148,149,150]. Although each antioxidant assay estimates a precise antioxidant feature of a sample, the present systematic review uncovers the need for primary studies’ data obtained according to a certain unified, widely accepted methodology to measure the antioxidant activity of plant extracts in order to better estimate the value of stevia in the agri-food sector. The movement towards evidence-based scientific practices encourages scientists and other decision-makers to pay more attention to evidence in order to eliminate unsound or outdated practices and perform more effective and firmly grounded scientific research [151]. This meta-analysis, having exploited the large number of primary studies’ data available on aqueous, hydroalcoholic, and organic extracts, provides the first step towards evidence-based scientific practice by showing that stevia leaf extracts made with a water:ethanol (1:1) mixture or pure water can most efficiently extract polyphenols, accompanied by the highest antioxidant activity. Only if an agreed set of protocols to measure antioxidant activity is followed could we compare the head-to-head effect sizes of the assays and infer the antioxidant value of stevia extracts. Moreover, our methodological approach can be applied to other plant extracts to uncover other specific features inherent to plant products and lead to a better understanding of the nature of the health benefits of medicinal plants.

## Figures and Tables

**Table 1:** Characteristics of the 250 studies included in the meta-analysis.

Author	Publication Year	Specific Study Characteristics	Country of Study	Assay	Measuring Unit	Assay Values	SEM	Extract/Chemical Compounds Group
Andrade et al. [120]	2021		Brazil	TPC	mg GAE/g	110.36	3.08	Aqueous
Andrade et al. [120]	2021		Brazil	TPC	mg GAE/g	78.00	0.87	Hydroalcoholic/12% ethanol
Chaiyana et al. [93]	2021		Thailand	TPC	mg GAE/g	9.40	0.12	Aqueous
Gullian-Klanian et al. [127]	2021		Canada	TPC	mg GAE/g	10.39	0.13	Hydroalcoholic/80% methanol
Mahajan et al. [122]	2021	2016	India	TPC	mg GAE/g	16.75	0.24	Hydroalcoholic/80% methanol
Mahajan et al. [122]	2021	2017	India	TPC	mg GAE/g	18.25	0.29	Hydroalcoholic/80% methanol
Tavarini et al. [126]	2020		Italy	TPC	mg GAE/g	74.88	1.73	Aqueous
Ahmad et al. [85]	2020		China	TPC	mg GAE/g	6.97	1.65	Organic/DMSO
Zorzenon et al. [106]	2020		Brazil	TPC	mg GAE/g	72.21	0.72	Hydroalcoholic/70% ethanol
Dyduch-Siemińska et al. [95]	2020	China	Poland	TPC	mg GAE/g	22.28	0.01	Organic/methanol
Dyduch-Siemińska et al. [95]	2020	Poland	Poland	TPC	mg GAE/g	16.14	0.07	Organic/methanol
Dyduch-Siemińska et al. [95]	2020	India	Poland	TPC	mg GAE/g	26.27	0.04	Organic/methanol
Dyduch-Siemińska et al. [95]	2020	Malaysia	Poland	TPC	mg GAE/g	26.27	0.05	Organic/methanol
Dyduch-Siemińska et al. [95]	2020	Paraguay	Poland	TPC	mg GAE/g	27.81	0.03	Organic/methanol
Dyduch-Siemińska et al. [95]	2020	Morocco	Poland	TPC	mg GAE/g	14.91	0.24	Organic/methanol
Dyduch-Siemińska et al. [95]	2020	Egypt	Poland	TPC	mg GAE/g	15.88	0.11	Organic/methanol
Dyduch-Siemińska et al. [95]	2020	Pakistan	Poland	TPC	mg GAE/g	25.25	0.04	Organic/methanol
Dyduch-Siemińska et al. [95]	2020	Australia	Poland	TPC	mg GAE/g	24.61	0.01	Organic/methanol
Dyduch-Siemińska et al. [95]	2020	Nigeria	Poland	TPC	mg GAE/g	15.83	0.09	Organic/methanol
Kovačević et al. [92]	2018	100 °C	Croatia	TPC	mg GAE/g	7.50	0.60	Aqueous
Kovačević et al. [92]	2018	130 °C	Croatia	TPC	mg GAE/g	7.90	0.16	Aqueous
Kovačević et al. [92]	2018	160 °C	Croatia	TPC	mg GAE/g	7.74	0.96	Aqueous
Atas et al. [87]	2017		Turkey	TPC	mg GAE/g	105.00	3.80	Organic/methanol
Barroso et al. [88]	2018	Frozen—dried	Portugal	TPC	mg GAE/g	85.17	0.33	Hydroalcoholic/80% methanol
Barroso et al. [88]	2018	Dried	Portugal	TPC	mg GAE/g	126.65	1.22	Hydroalcoholic/80% methanol
Formigoni et al. [98]	2018		Brazil	TPC	mg GAE/g	5.54	0.09	Aqueous
S. Latha et al. [114]	2017		India	TPC	mg GAE/g	126.50	1.24	Hydroalcoholic/50% methanol
Milani et al. [105]	2017		Brazil	TPC	mg GAE/g	23.61	0.10	Aqueous
Milani et al. [105]	2017	EtOH—maceration	Brazil	TPC	mg GAE/g	4.456	0.01	Organic/ethanol
Milani et al. [105]	2017	EtOH—soxhlet	Brazil	TPC	mg GAE/g	30.267	0.06	Organic/ethanol
Milani et al. [105]	2017	MeOH—soxhlet	Brazil	TPC	mg GAE/g	41.63	0.19	Organic/methanol
Milani et al. [128]	2017		Brazil	TPC	mg GAE/g	187.66	0.11	Phenols/fraction
Yu et al. [107]	2017		China	TPC	mg GAE/g	71.46	1.44	Aqueous
Moselhy et al. [123]	2016		Saudi Arabia	TPC	mg GAE/g	30.00	0.87	Aqueous
Moselhy et al. [123]	2016		Saudi Arabia	TPC	mg GAE/g	85.00	1.44	Organic/acetone
Torri et al. [108]	2017		Italy	TPC	mg GAE/g	76.60	3.93	Aqueous
Javed et al. [102]	2017		Italy	TPC	mg GAE/g	4.52	0.02	Organic/acetone
Ruiz-Ruiz et al. [115]	2015		Mexico	TPC	mg GAE/g	28.40	0.87	Aqueous
Tavarini et al. [110]	2015	Harvest time—9 Jul	Italy	TPC	mg GAE/g	75.00	5.77	Hydroalcoholic/70% ethanol
Tavarini et al. [110]	2015	Harvest time—21 Jul	Italy	TPC	mg GAE/g	40.00	0.58	Hydroalcoholic/70% ethanol
Tavarini et al. [110]	2015	Harvest time—10 Sep	Italy	TPC	mg GAE/g	70.00	0.58	Hydroalcoholic/70% ethanol
Bender et al. [89]	2015	Powder-leaf	Italy	TPC	mg GAE/g	17.49	1.39	Aqueous
Bender et al. [89]	2015	Powder-leaf-organic	Italy	TPC	mg GAE/g	89.49	1.44	Aqueous
Bender et al. [89]	2015	Leaf-company1	Italy	TPC	mg GAE/g	195.30	2.40	Aqueous
Bender et al. [89]	2015	Leaf-company2	Italy	TPC	mg GAE/g	149.06	6.58	Aqueous
Bender et al. [89]	2015	Leaf-Peru	Italy	TPC	mg GAE/g	60.19	1.44	Aqueous
Gaweł-Bęben et al. [99]	2015	Aqueous	Poland	TPC	mg GAE/g	3.85	0.06	Flavonoids/fraction
Gaweł-Bęben et al. [99]	2015	Ethanol	Poland	TPC	mg GAE/g	7.65	0.06	Flavonoids/fraction
Gaweł-Bęben et al. [99]	2015	Glycol–aqueous	Poland	TPC	mg GAE/g	15.50	0.23	Flavonoids/fraction
Periche et al. [119]	2015	90 °C	Spain	TPC	mg GAE/g	93.41	2.89	Aqueous
Periche et al. [119]	2015	50 °C	Spain	TPC	mg GAE/g	80.00	1.44	Aqueous
Periche et al. [119]	2015	Microwave	Spain	TPC	mg GAE/g	81.00	3.35	Aqueous
Periche et al. [118]	2015	Drying—180 °C	Spain	TPC	mg GAE/g	76.80	1.02	Hydroalcoholic/60% ethanol
Periche et al. [118]	2015	Shade-drying	Spain	TPC	mg GAE/g	39.10	0.61	Hydroalcoholic/60% ethanol
Periche et al. [118]	2015	Drying—100 °C	Spain	TPC	mg GAE/g	31.50	0.41	Hydroalcoholic/60% ethanol
Periche et al. [118]	2015	Freeze-drying	Spain	TPC	mg GAE/g	26.20	1.14	Hydroalcoholic/60% ethanol
Ruiz et al. [116]	2015	MoritaII	Mexico	TPC	mg GAE/g	28.70	0.88	Organic/methanol
Ruiz et al. [116]	2015	Criolla	Mexico	TPC	mg GAE/g	28.40	0.87	Organic/methanol
Periche et al. [117]	2014	50 °C	Spain	TPC	mg GAE/g	65.20	1.15	Aqueous
Periche et al. [117]	2014	70 °C	Spain	TPC	mg GAE/g	71.02	1.21	Aqueous
Periche et al. [117]	2014	90 °C	Spain	TPC	mg GAE/g	92.07	1.15	Aqueous
Periche et al. [117]	2014	1 min	Spain	TPC	mg GAE/g	71.70	2.25	Aqueous
Periche et al. [117]	2014	5 min	Spain	TPC	mg GAE/g	75.71	1.73	Aqueous
Periche et al. [117]	2014	20 min	Spain	TPC	mg GAE/g	77.34	2.02	Aqueous
Periche et al. [117]	2014	40 min	Spain	TPC	mg GAE/g	79.63	2.05	Aqueous
Tavarini et al. [109]	2013	Jesi-2009	Italy	TPC	mg GAE/g	52.23	2.25	Organic/methanol
Tavarini et al. [109]	2013	PieveCesato-2009	Italy	TPC	mg GAE/g	48.12	3.97	Organic/methanol
Tavarini et al. [109]	2013	Jesi-2010	Italy	TPC	mg GAE/g	66.90	5.83	Organic/methanol
Tavarini et al. [109]	2013	PieveCesato-2010	Italy	TPC	mg GAE/g	54.91	4.66	Organic/methanol
Shivanna et al. [113]	2013		India	TPC	mg GAE/g	91.00	1.77	Organic/methanol
Shukla et al. [111]	2012		India	TPC	mg GAE/g	56.73	2.25	Aqueous
Shukla et al. [111]	2009		India	TPC	mg GAE/g	2.77	0.10	Organic/ethanol
Alshawwa et al. [86]	2022	Chloroform–methanol	Saudi Arabia	TPC	mg GAE/g	26.25	0.12	Organic/chloroform–methanol
Alshawwa et al. [86]	2022	Acetone	Saudi Arabia	TPC	mg GAE/g	2.38	0.04	Organic/acetone
Alshawwa et al. [86]	2022	Ethyl acetate	Saudi Arabia	TPC	mg GAE/g	18.00	0.09	Organic/ethyl acetate
El-Hadary et al. [96]	2021		Egypt	TPC	mg GAE/g	12.80	0.36	Hydroalcoholic/80% ethanol
Judickaitė et al. [103]	2022		Lithuania	TPC	mg GAE/g	13.00	0.05	Hydroalcoholic/70% ethanol
Sheikhalipour et al. [125]	2021		Iran	TPC	mg GAE/g	30.50	0.50	Hydroalcoholic/85% methanol
Oviedo-Pereira et al. [124]	2022		Mexico	TPC	mg GAE/g	20.00	1.39	Hydroalcoholic /75% ethanol
Elsayed et al. [97]	2022		Egypt	TPC	mg GAE/g	21.50	0.75	Organic/methanol
Bugliani et al. [91]	2022	Chemotype-1	Italy	TPC	mg GAE/g	155.00	6.60	Hydroalcoholic/50% ethanol
Bugliani et al. [91]	2022	Chemotype-2	Italy	TPC	mg GAE/g	175.95	2.40	Hydroalcoholic/50% ethanol
Bugliani et al. [91]	2022	Chemotype-3	Italy	TPC	mg GAE/g	117.72	2.88	Hydroalcoholic/50% ethanol
Bugliani et al. [91]	2022	Chemotype-4	Italy	TPC	mg GAE/g	93.39	4.17	Hydroalcoholic/50% ethanol
Bugliani et al. [91]	2022	Chemotype-5	Italy	TPC	mg GAE/g	63.01	2.48	Hydroalcoholic/50% ethanol
Bugliani et al. [91]	2022	Chemotype-6	Italy	TPC	mg GAE/g	64.39	0.50	Hydroalcoholic/50% ethanol
Judickaitė et al. [104]	2023		Lithuania	TPC	mg GAE/g	81.07	9.37	Hydroalcoholic/70% ethanol
Kaushik et al. [121]	2010		India	TPC	mg TAE/g	42.00	5.77	Organic/acetonitrile
Tavarini et al. [126]	2020		Italy	TFC	mg CE/g	71.13	1.38	Aqueous
Tavarini et al. [110]	2015	Harvest time—9 Jul	Italy	TFC	mg CE/g	70.00	3.61	Hydroalcoholic/70% ethanol
Tavarini et al. [110]	2015	Harvest time—21 Jul	Italy	TFC	mg CE/g	31.00	6.99	Hydroalcoholic/70% ethanol
Tavarini et al. [110]	2015	Harvest time—10 Sep	Italy	TFC	mg CE/g	71.00	4.68	Hydroalcoholic/70% ethanol
Periche et al. [119]	2015	90 °C	Spain	TFC	mg CE/g	58.00	2.94	Aqueous
Periche et al. [119]	2015	50 °C	Spain	TFC	mg CE/g	43.00	1.62	Aqueous
Periche et al. [119]	2015	Microwave	Spain	TFC	mg CE/g	45.00	4.33	Aqueous
Periche et al. [118]	2015	Drying—180 °C	Spain	TFC	mg CE/g	45.10	0.61	Hydroalcoholic/60% ethanol
Periche et al. [118]	2015	Shade-drying	Spain	TFC	mg CE/g	20.30	0.31	Hydroalcoholic/60% ethanol
Periche et al. [118]	2015	Drying—100 °C	Spain	TFC	mg CE/g	17.20	0.20	Hydroalcoholic/60% ethanol
Periche et al. [118]	2015	Freeze-drying	Spain	TFC	mg CE/g	9.90	0.20	Hydroalcoholic/60% ethanol
Periche et al. [117]	2014	50 °C	Spain	TFC	mg CE/g	32.47	1.15	Aqueous
Periche et al. [117]	2014	70 °C	Spain	TFC	mg CE/g	47.53	1.04	Aqueous
Periche et al. [117]	2014	90 °C	Spain	TFC	mg CE/g	56.72	1.21	Aqueous
Periche et al. [117]	2014	1 min	Spain	TFC	mg CE/g	44.64	0.58	Aqueous
Periche et al. [117]	2014	5 min	Spain	TFC	mg CE/g	45.93	1.01	Aqueous
Periche et al. [117]	2014	20 min	Spain	TFC	mg CE/g	45.77	1.07	Aqueous
Periche et al. [117]	2014	40 min	Spain	TFC	mg CE/g	44.26	1.04	Aqueous
Bugliani et al. [91]	2022	Chemotype-1	Italy	TFC	mg CE/g	128.08	0.81	Hydroalcoholic/50% ethanol
Bugliani et al. [91]	2022	Chemotype-2	Italy	TFC	mg CE/g	150.24	4.57	Hydroalcoholic/50% ethanol
Bugliani et al. [91]	2022	Chemotype-3	Italy	TFC	mg CE/g	105.34	5.80	Hydroalcoholic/50% ethanol
Bugliani et al. [91]	2022	Chemotype-4	Italy	TFC	mg CE/g	39.46	0.94	Hydroalcoholic/50% ethanol
Bugliani et al. [91]	2022	Chemotype-5	Italy	TFC	mg CE/g	31.04	0.09	Hydroalcoholic/50% ethanol
Bugliani et al. [91]	2022	Chemotype-6	Italy	TFC	mg CE/g	28.32	0.97	Hydroalcoholic/50% ethanol
Andrade et al. [120]	2021		Brazil	TFC	mg QE/g	71.79	0.01	Aqueous
Andrade et al. [120]	2021		Brazil	TFC	mg QE/g	56.66	0.53	Hydroalcoholic/12% ethanol
Chaiyana et al. [93]	2021		Thailand	TFC	mg QE/g	8.10	0.06	Aqueous
Gullian-Klanian et al. [127]	2021		Canada	TFC	mg QE/g	71.30	1.10	Hydroalcoholic/80% ethanol
Ahmad et al. [85]	2020		China	TFC	mg QE/g	4.44	0.90	Organic/DMSO
Atas et al. [87]	2017		Turkey	TFC	mg QE/g	95.00	2.37	Organic/methanol
Formigoni et al. [98]	2018		Brazil	TFC	mg QE/g	1.53	0.05	Aqueous
Milani et al. [105]	2017		Brazil	TFC	mg QE/g	176.33	10.00	Aqueous
Milani et al. [105]	2017	EtOH—maceration	Brazil	TFC	mg QE/g	17.16	1.22	Organic/ethanol
Milani et al. [105]	2017	EtOH—Soxhlet	Brazil	TFC	mg QE/g	19.60	0.10	Organic/ethanol
Milani et al. [105]	2017	MeOH—Soxhlet	Brazil	TFC	mg QE/g	21.45	3.06	Organic/methanol
Milani et al. [128]	2017		Brazil	TFC	mg QE/g	0.14	0.04	Phenols/fraction
Moselhy et al. [123]	2016		Saudi Arabia	TFC	mg QE/g	40.00	2.94	Aqueous
Moselhy et al. [123]	2016		Saudi Arabia	TFC	mg QE/g	80.00	4.68	Organic/acetone
Javed et al. [102]	2017		Italy	TFC	mg QE/g	3.81	0.04	Organic/acetone
Ruiz-Ruiz et al. [115]	2015		Mexico	TFC	mg QE/g	36.70	2.94	Aqueous
Gaweł-Bęben et al. [99]	2015	Phenols	Poland	TFC	mg QE/g	2.03	0.06	Phenols/fraction
Gaweł-Bęben et al. [99]	2015	Phenols	Poland	TFC	mg QE/g	2.25	0.03	Phenols/fraction
Gaweł-Bęben et al. [99]	2015	Phenols	Poland	TFC	mg QE/g	3.85	0.06	Phenols/fraction
Ruiz et al. [116]	2015	MoritaII	Mexico	TFC	mg QE/g	39.30	3.00	Organic/methanol
Ruiz et al. [116]	2015	Criolla	Mexico	TFC	mg QE/g	36.70	2.94	Organic/methanol
Shivanna et al. [113]	2013		India	TFC	mg QE/g	23.00	1.13	Organic/methanol
El-Hadary et al. [96]	2021		Egypt	TFC	mg QE/g	2.31	0.31	Hydroalcoholic 80% ethanol
Oviedo-Pereira et al. [124]	2022		Mexico	TFC	mg QE/g	16.25	1.21	Hydroalcoholic 75% ethanol
Elsayed et al. [97]	2022		Egypt	TFC	mg QE/g	3.60	0.10	Organic/methanol
Dyduch-Siemińska et al. [95]	2020	China	Poland	TFC	mg RUE/g	10.19	0.11	Organic/methanol
Dyduch-Siemińska et al. [95]	2020	Poland	Poland	TFC	mg RUE/g	12.94	0.19	Organic/methanol
Dyduch-Siemińska et al. [95]	2020	India	Poland	TFC	mg RUE/g	6.60	0.29	Organic/methanol
Dyduch-Siemińska et al. [95]	2020	Malaysia	Poland	TFC	mg RUE/g	6.51	0.10	Organic/methanol
Dyduch-Siemińska et al. [95]	2020	Paraguay	Poland	TFC	mg RUE/g	5.87	0.14	Organic/methanol
Dyduch-Siemińska et al. [95]	2020	Morocco	Poland	TFC	mg RUE/g	12.99	0.23	Organic/methanol
Dyduch-Siemińska et al. [95]	2020	Egypt	Poland	TFC	mg RUE/g	12.50	0.14	Organic/methanol
Dyduch-Siemińska et al. [95]	2020	Pakistan	Poland	TFC	mg RUE/g	7.32	0.04	Organic/methanol
Dyduch-Siemińska et al. [95]	2020	Australia	Poland	TFC	mg RUE/g	9.18	0.05	Organic/methanol
Dyduch-Siemińska et al. [95]	2020	Nigeria	Poland	TFC	mg RUE/g	12.38	0.16	Organic/methanol
S. Latha et al. [114]	2017		India	TFC	mg RUE/g	128.30	5.02	Hydroalcoholic/50% ethanol
Torri et al. [108]	2017		Italy	TFC	mg RUE/g	34.30	2.94	Aqueous
Judickaitė et al. [103]	2022		Lithuania	TFC	mg RUE/g	4.30	0.01	Hydroalcoholic/70% ethanol
Judickaitė et al. [103]	2023		Lithuania	TFC	mg RUE/g	56.17	2.14	Hydroalcoholic/70% ethanol
El-Hadary et al. [96]	2021		Egypt	ABTS	%	42.33	0.55	Hydroalcoholic/80% ethanol
Andrade et al. [120]	2021		Brazil	ABTS	µmol TE/g	680.00	5.77	Aqueous
Andrade et al. [120]	2021		Brazil	ABTS	µmol TE/g	660.00	34.64	Hydroalcoholic/12% ethanol
Gullian-Klanian et al. [127]	2021		Canada	ABTS	µmol TE/g	510.90	2.50	Hydroalcoholic/80% methanol
Ruiz et al. [116]	2015	MoritaII	Mexico	ABTS	µmol TE/g	311.20	10.00	Organic/methanol
Ruiz et al. [116]	2015	Criolla	Mexico	ABTS	µmol TE/g	316.60	11.00	Organic/methanol
Chaiyana et al. [93]	2021		Thailand	DPPH	%	89.00	0.58	Aqueous
Gullian-Klanian et al. [127]	2021		Canada	DPPH	%	19.50	1.30	Hydroalcoholic
Ahmad et al. [85]	2020		China	DPPH	%	48.03	2.50	Organic
Zorzenon et al. [106]	2020		Brazil	DPPH	%	87.47	0.11	Hydroalcoholic
Dyduch-Siemińska et al. [95]	2020	China	Poland	DPPH	%	24.04	0.17	Organic
Dyduch-Siemińska et al. [95]	2020	Poland	Poland	DPPH	%	48.16	0.29	Organic
Dyduch-Siemińska et al. [95]	2020	India	Poland	DPPH	%	21.05	0.08	Organic
Dyduch-Siemińska et al. [95]	2020	Malaysia	Poland	DPPH	%	32.69	0.30	Organic
Dyduch-Siemińska et al. [95]	2020	Paraguay	Poland	DPPH	%	29.10	0.27	Organic
Dyduch-Siemińska et al. [95]	2020	Morocco	Poland	DPPH	%	43.25	0.33	Organic
Dyduch-Siemińska et al. [95]	2020	Egypt	Poland	DPPH	%	47.04	0.24	Organic
Dyduch-Siemińska et al. [95]	2020	Pakistan	Poland	DPPH	%	20.58	0.08	Organic
Dyduch-Siemińska et al. [95]	2020	Australia	Poland	DPPH	%	20.23	0.11	Organic
Dyduch-Siemińska et al. [95]	2020	Nigeria	Poland	DPPH	%	45.08	0.25	Organic
Atas et al. [87]	2017		Turkey	DPPH	%	35.00	0.33	Organic
Formigoni et al. [98]	2018		Brazil	DPPH	%	88.72	0.66	Aqueous
S. Latha et al. [114]	2017		India	DPPH	%	77.13	1.00	Hydroalcoholic
S. Latha et al. [114]	2017		India	DPPH	%	67.23	1.00	Glycosides/stevioside
Milani et al. [105]	2017		Brazil	DPPH	%	42.26	0.64	Aqueous
Milani et al. [105]	2017	EtOH—maceration	Brazil	DPPH	%	80.00	1.00	Organic
Milani et al. [105]	2017	EtOH—Soxhlet	Brazil	DPPH	%	87.00	1.00	Organic
Milani et al. [105]	2017	MeOH—Soxhlet	Brazil	DPPH	%	93.00	1.00	Organic
Tavarini et al. [109]	2013	Jesi-2009	Italy	DPPH	%	87.61	0.66	Organic
Tavarini et al. [109]	2013	PieveCesato-2009	Italy	DPPH	%	87.12	0.47	Organic
Tavarini et al. [109]	2013	Jesi-2010	Italy	DPPH	%	88.84	0.47	Organic
Tavarini et al. [109]	2013	PieveCesato-2010	Italy	DPPH	%	88.41	0.45	Organic
Shukla et al. [111]	2012		India	DPPH	%	72.37	1.00	Aqueous
Shukla et al. [112]	2009		India	DPPH	%	68.76	0.64	Organic
El-Hadary et al. [96]	2021		Egypt	DPPH	%	44.15	0.64	Hydroalcoholic
Elsayed et al. [97]	2022		Egypt	DPPH	%	78.00	1.00	Organic
Ahmad et al. [85]	2020		China	DPPH	mg AAE/g	13.70	1.73	Organic
Tavarini et al. [110]	2015	Harvest time—9 Jul	Italy	DPPH	mg AAE/g	14.44	0.15	Hydroalcoholic
Tavarini et al. [110]	2015	Harvest time—21 Jul	Italy	DPPH	mg AAE/g	14.09	0.13	Hydroalcoholic
Tavarini et al. [110]	2015	Harvest time—10 Sep	Italy	DPPH	mg AAE/g	14.26	0.14	Hydroalcoholic
Elsayed et al. [97]	2022		Egypt	DPPH	mg AAE/g	3.50	0.50	Organic
Judickaitė et al. [103]	2022		Lithuania	DPPH	mg RUE/g	48.00	0.10	Hydroalcoholic
Judickaitė et al. [104]	2023		Lithuania	DPPH	mg RUE/g	32.15	0.93	Hydroalcoholic
Gullian-Klanian et al. [127]	2021		Canada	DPPH	mg TE/g	24.49	0.51	Hydroalcoholic
Tavarini et al. [126]	2020		Italy	DPPH	mg TE/g	40.30	0.29	Aqueous
Periche et al. [119]	2015	90 °C	Spain	DPPH	mg TE/g	131.00	2.89	Aqueous
Periche et al. [119]	2015	50 °C	Spain	DPPH	mg TE/g	81.00	5.77	Aqueous
Periche et al. [119]	2015	Microwave	Spain	DPPH	mg TE/g	96.00	2.60	Aqueous
Periche et al. [118]	2015	Drying at 180 °C	Spain	DPPH	mg TE/g	126.00	0.31	Hydroalcoholic
Periche et al. [118]	2015	Shade-drying	Spain	DPPH	mg TE/g	75.90	0.05	Hydroalcoholic
Periche et al. [118]	2015	Drying at 100 °C	Spain	DPPH	mg TE/g	64.90	0.33	Hydroalcoholic
Periche et al. [118]	2015	Freeze-drying	Spain	DPPH	mg TE/g	48.50	0.29	Hydroalcoholic
Periche et al. [117]	2014	50 °C	Spain	DPPH	mg TE/g	62.45	0.46	Aqueous
Periche et al. [117]	2014	70 °C	Spain	DPPH	mg TE/g	106.68	0.43	Aqueous
Periche et al. [117]	2014	90 °C	Spain	DPPH	mg TE/g	119.12	0.43	Aqueous
Periche et al. [117]	2014	1 min	Spain	DPPH	mg TE/g	87.96	2.60	Aqueous
Periche et al. [117]	2014	5 min	Spain	DPPH	mg TE/g	89.26	2.60	Aqueous
Periche et al. [117]	2014	20 min	Spain	DPPH	mg TE/g	102.64	2.89	Aqueous
Periche et al. [117]	2014	40 min	Spain	DPPH	mg TE/g	104.48	2.89	Aqueous
Chaiyana et al. [93]	2021		Thailand	FRAP	μmol Fe^2+^/g	210.00	2.89	Aqueous
Tavarini et al. [109]	2013	Jesi-2009	Italy	FRAP	μmol Fe^2+^/g	495.54	107.7	Organic/methanol
Tavarini et al. [109]	2013	PieveCesato-2009	Italy	FRAP	μmol Fe^2+^/g	471.70	24.18	Organic/methanol
Tavarini et al. [109]	2013	Jesi-2010	Italy	FRAP	μmol Fe^2+^/g	690.33	75.95	Organic/methanol
Tavarini et al. [109]	2013	PieveCesato-2010	Italy	FRAP	μmol Fe^2+^/g	621.89	62.19	Organic/methanol
Devi et al. [94]	2023		India	FRAP	μmol Fe^2+^/g	154.79	0.69	Organic/methanol
Devi et al. [94]	2023		India	FRAP	μmol Fe^2+^/g	149.64	1.73	Organic/chloroform
Andrade et al. [120]	2021		Brazil	FRAP	μmol ΤΕ/g	220.00	5.77	Aqueous
Andrade et al. [120]	2021		Brazil	FRAP	μmol ΤΕ/g	230.00	1.73	Hydroalcoholic/12% ethanol
Tavarini et al. [126]	2020		Italy	FRAP	μmol ΤΕ/g	532.00	11.55	Aqueous
Tavarini et al. [110]	2015	Harvest time—9 Jul	Italy	FRAP	μmol ΤΕ/g	580.00	7.51	Hydroalcoholic/70% ethanol
Tavarini et al. [110]	2015	Harvest time—21 Jul	Italy	FRAP	μmol ΤΕ/g	251.00	18.48	Hydroalcoholic/70% ethanol
Tavarini et al. [110]	2015	Harvest time—10 Sep	Italy	FRAP	μmol ΤΕ/g	460.00	15.01	Hydroalcoholic/70% ethanol
Bugliani et al. [91]	2022	Chemotype-1	Italy	FRAP	μmol ΤΕ/g	1510.00	80.00	Hydroalcoholic/70% ethanol
Bugliani et al. [91]	2022	Chemotype-2	Italy	FRAP	μmol ΤΕ/g	2310.00	100.00	Hydroalcoholic/70% ethanol
Bugliani et al. [91]	2022	Chemotype-3	Italy	FRAP	μmol ΤΕ/g	1250.00	60.00	Hydroalcoholic/70% ethanol
Bugliani et al. [91]	2022	Chemotype-4	Italy	FRAP	μmol ΤΕ/g	940.00	30.00	Hydroalcoholic/70% ethanol
Bugliani et al. [91]	2022	Chemotype-5	Italy	FRAP	μmol ΤΕ/g	680.00	20.00	Hydroalcoholic/70% ethanol
Bugliani et al. [91]	2022	Chemotype-6	Italy	FRAP	μmol ΤΕ/g	590.00	10.00	Hydroalcoholic/70% ethanol
Andrade et al. [120]	2021		Brazil	ORAC	μmol ΤΕ/g	510.35	8.34	Aqueous
Andrade et al. [120]	2021		Brazil	ORAC	μmol ΤΕ/g	454.89	0.63	Hydroalcoholic/12% ethanol
Tavarini et al. [126]	2020		Italy	ORAC	μmol ΤΕ/g	2231.19	61.31	Aqueous
Torri et al. [108]	2017		Italy	ORAC	μmol ΤΕ/g	648.00	3.67	Aqueous
Lemus-Mondaca et al. [28]	2016		Chile	ORAC	μmol ΤΕ/g	222.59	4.60	Hydroalcoholic/50% ethanol
Bender et al. [89]	2015	Powder-leaf	Italy	ORAC	μmol ΤΕ/g	275.70	11.55	Aqueous
Bender et al. [89]	2015	Powder-leaf organic	Italy	ORAC	μmol ΤΕ/g	958.80	54.96	Aqueous
Bender et al. [89]	2015	Leaf-company1	Italy	ORAC	μmol ΤΕ/g	971.10	8.66	Aqueous
Bender et al. [89]	2015	Leaf-company2	Italy	ORAC	μmol ΤΕ/g	1040.00	25.98	Aqueous
Bender et al. [89]	2015	Leaf-Peru	Italy	ORAC	μmol ΤΕ/g	1071.00	4.62	Aqueous
Mahajan et al. [122]	2021	2016	India	SOD	U/g	79.00	0.50	Fresh leaf extract
Mahajan et al. [122]	2021	2017	India	SOD	U/g	104.00	1.00	Fresh leaf extract
Borbély et al. [90]	2021		Portugal	SOD	U/g	112.50	2.00	Fresh leaf extract
Sheikhalipour et al. [125]	2021		Iran	SOD	U/mg protein	3.30	0.05	Fresh leaf extract
Ghorbani et al. [101]	2023		China	SOD	U/mg protein	98.00	0.45	Fresh leaf extract
Mahajan et al. [122]	2021	2016	India	CAT	U/g	22.00	1.00	Fresh leaf extract
Gerami et al. [100]	2020		Iran	CAT	U/g	550.00	40.41	Fresh leaf extract
Elsayed et al. [97]	2022		Egypt	CAT	U/g	0.15	0.01	Fresh leaf extract
Sheikhalipour et al. [125]	2021		Iran	CAT	U/mg protein	5.50	0.10	Fresh leaf extract
Ghorbani et al. [101]	2023		China	CAT	U/mg protein	99.00	0.41	Fresh leaf extract
Mahajan et al. [122]	2021	2016	India	POX	U/g	21.00	1.00	Fresh leaf extract
Mahajan et al. [122]	2021	2017	India	POX	U/g	40.00	1.00	Fresh leaf extract
Elsayed et al. [97]	2022		Egypt	POX	U/g	0.34	0.01	Fresh leaf extract
Gerami et al. [100]	2020		Iran	MDA	mM/g	1.70	0.12	Fresh leaf extract
Sheikhalipour et al. [125]	2021		Iran	MDA	mM/g	1.80	0.15	Fresh leaf extract
Ghorbani et al. [101]	2023		China	MDA	mM/g	2.35	0.12	Fresh leaf extract

TPC: total phenolic content; TFC: total flavonoid content; ABTS: {2,2′–azinobis-(3-ethyl-benzothiazoline-6-sulphonicacid)}; DPPH: 2,2-Diphenyl-1-picrylhydrazyl; FRAP: ferric reducing antioxidant power; ORAC: oxygen radical absorbance capacity; SOD: superoxide dismutase; CAT: catalase; POX: peroxidase; MDA: malondialdehyde; GAE: gallic acid equivalent; TAE: tannic acid equivalent; CE: catechin equivalent; QE: quercetin equivalent; RUE: rutin equivalent; ΤΕ: Trolox equivalent; EtOH: ethanol; Hydroalcoholic: a solvent mixture of water and alcohol.

**Table 2:** Stratification meta-analysis according to the type of stevia extract for the TPC assay.

Assay	Type of Extract	Effect Size	CI 95%	Number of Studies
TPC (mg GAE/g)	Aqueous	63.73	58.37, 69.09	28
	Hydroalcoholic	64.77	56.39, 73.15	27
	Organic	33.39	28.75, 38.02	26
	Phenols	187.66	187.45, 187.87	1
	Flavonoids	8.99	5.02, 12.95	3
Hydroalcoholic (methanol/ethanol)	Methanol	47.93	24.72, 71.13	6
	Ethanol	71.26	58.16, 84.37	20
Hydroalcoholic (alcohol %)	Methanol 80%	51.41	24.92, 77.91	5
	Methanol 85%	30.50	29.52, 31.48	1
	Ethanol 12%	78.00	76.29, 79.70	1
	Ethanol 50%	113.60	79.20, 148.01	7
	Ethanol 60%	43.39	26.55, 60.23	4
	Ethanol 70%	58.20	30.50, 85.90	6
	Ethanol 75%	20.00	17.27, 22.72	1
	Ethanol 80%	12.80	12.09, 13.50	1
Organic	Methanol	30.58	29.34, 31.82	20
	Ethanol	12.49	0.54, 30.38	3
	Acetone	25.12	22.35, 27.89	3
	Acetonitrile	42.00	30.69, 53.30	1
	Chloroform–methanol	26.25	26.01, 26.48	1
	Ethyl acetate	18.00	17.82, 18.17	1
	DMSO	6.97	3.73, 10.20	1

CI 95%: 95% confidence interval; GAE: gallic acid equivalent; Hydroalcoholic: a solvent mixture of water and alcohol.

**Table 3:** Stratification meta-analysis according to the type of stevia extract for the TFC assay.

Assay	Type of Extract	Effect Size	CI 95%	Number of Studies
TFC all units	Aqueous	49.72	27.89, 71.55	18
	Hydroalcoholic	53.41	45.17, 61.64	20
	Organic	17.80	15.89, 19.71	21
	Phenols	2.067	0.623, 3.511	4
TFC mg QE/g	Aqueous	54.90	16.78, 93.02	6
	Hydroalcoholic	36.62	0.66, 72.59	4
	Organic	29.78	24.57, 34.99	11
	Phenols	2.07	0.62, 3.51	4
TFC mg CE/g	Aqueous	44.26	42.22, 46.29	11
	Hydroalcoholic	56.75	47.33, 66.17	13
	Organic	nr*		
	Phenols	nr*		
TFC mg RUE/g	Aqueous	34.30	28.53, 40.06	1
	Hydroalcoholic	62.69	8.11, 117.27	3
	Organic	9.64	8.34, 10.95	10
	Phenols	nr*		
TFC all units				
Hydroalcoholic (methanol/ethanol)	Methanol	71.30	69.14, 73.45	1
	Ethanol	52.43	44.06, 60.79	19
Hydroalcoholic (alcohol %)	Methanol 80%	71.30	69.14, 73.45	1
	Methanol 85%	nr*		
	Ethanol 12%	56.66	55.62, 57.69	1
	Ethanol 50%	87.05	53.39, 120.71	7
	Ethanol 60%	23.10	14.18, 32.02	4
	Ethanol 70%	46.47	11.57, 81.37	5
	Ethanol 75%	16.25	13.87, 18.62	1
	Ethanol 80%	2.31	1.70, 2.91	1
Organic	Methanol	15.83	14.39, 17.27	16
	Ethanol	18.68	16.36, 20.99	2
	DMSO	4.44	2.67, 6.20	1

nr*: not reported; CI 95%: 95% confidence interval; QE: quercetin equivalent; CE: catechin equivalent; RUE: rutin equivalent; Hydroalcoholic: a solvent mixture of water and alcohol.

**Table 4:** Stratification meta-analysis of data on individual phenolic compound concentrations according to the type of stevia extract.

Type of Extract	Compound	Effect Size (mg CGAE/g dry sample)	CI 95%	Number of Studies
Aqueous	3-caffeoylquinic acid	2.25	1.24, 3.26	5
	3,4-dicaffeoylquinic acid	2.64	1.16, 4.11	5
	3,5-dicaffeoylquinic acid	50.53	25.21, 75.82	5
	4-caffeoylquinic acid	11.85	6.09, 17.60	5
	4,5-dicaffeoylquinic acid	18.69	4.73, 32.63	5
	5-caffeoylquinic acid	1.65	0.69, 2.58	5
	5-coumaroyl quinic acid	0.07	0.05, 0.09	5
	caffeoyl shikimic acid	0.36	0.12, 0.59	5
	**Compound**	**Effect Size** **(mg/g dry sample)**	**CI 95%**	**Number of Studies**
Hydroalcoholic	3-caffeoylquinic acid	2.91	0.70, 5.11	4
Organic	4,5-dicaffeoylquinic acid	13.15	9.20, 17.11	5
	5-caffeoylquinic acid	23.39	19.44, 27.34	5

CGAE: chlorogenic acid equivalents; CI 95%: 95% confidence interval.

**Table 5:** Stratification meta-analysis according to the type of stevia extract for the DPPH assay.

Assay	Type of Extract	Effect Size	CI 95%	Number of Studies
DPPH % RSA	Aqueous	73.09	49.55, 96.63	4
	Hydroalcoholic	72.31	52.96, 91.67	4
	Organic	55.84	48.85, 62.83	21
	Glycosides	67.23	65.27, 69.19	1
DPPH mg/g, all units	Aqueous	92.80	69.46, 116.14	11
	Hydroalcoholic	46.27	25.45, 67.10	10
	Organic	8.47	0.15, 18.46	2
DPPH mg TE/g	Aqueous	92.80	69.46, 12.14	11
	Hydroalcoholic	67.96	44.93, 90.99	5
	Organic	nr*		
DPPPH mg AAE/g	Aqueous	nr*		
	Hydroalcoholic	14.25	14.05, 14.45	3
	Organic	8.47	0.15, 18.46	2
DPPH mg RUE/g	Aqueous	nr*		
	Hydroalcoholic	40.10	24.57, 55.63	2
	Organic	nr*		

nr*: not reported; CI 95%: 95% confidence interval; RSA: radical scavenging activity; TE: Trolox equivalent; AAE: ascorbic acid equivalent; RUE: rutin equivalent; Hydroalcoholic: a solvent mixture of water and alcohol.

**Table 6:** Stratification meta-analysis according to the type of stevia extract for FRAP, ABTS, and ORAC assays.

Assay	Type of Extract	Effect Size	CI 95%	Number of Studies
FRAP all units	Aqueous	320.02	194.79, 445.23	3
	Hydroalcoholic	855.54	687.10, 1024.0	10
	Organic	248.40	221.63, 275.17	6
FRAP µmol Fe^+2^/g	Aqueous	210.00	204.33, 215.66	1
	Hydroalcoholic	nr*		
	Organic	248.40	221.63, 275.17	6
FRAP µmol TE/g	Aqueous	375.84	70.08, 681.59	2
	Hydroalcoholic	855.53	687.1, 1023.9	10
	Organic	nr*		
FRAP hydroalcoholic	Ethanol 12%	230.00	226.61, 233.39	1
(alcohol %)	Ethanol 50%	1192.51	943.09, 1441.9	6
	Ethanol 70%	430.93	250.17, 611.71	3
ABTS μmol TE/g	Aqueous	680.00	668.69, 691.30	1
	Hydroalcoholic	581.44	435.54, 727.35	2
	Organic	313.64	299.14, 328.14	2
ORAC µmol TE/g	Aqueous	879.28	677.37, 1081.2	8
	Hydroalcoholic	454.90	453.65, 456.12	2
	Organic	nr*		
**Meta-analysis of FRAP, ABTS, and ORAC**				
	Aqueous	769.20	587.98, 950.43	11
	Hydroalcoholic	720.48	645.31, 795.65	14
	Organic	313.64	299.14, 328.15	2

nr*: not reported; CI 95%: 95% confidence interval; TE: Trolox equivalent; Hydroalcoholic: a solvent mixture of water and alcohol.

**Table 7:** Meta-analysis of SOD, CAT, POX, and MDA assays data.

Assay	Measuring Unit	Effect Size	CI 95%	Number of Studies
SOD	U/g	98.44	77.35, 119.53	3
CAT	U/g	41.49	26.75, 56.23	3
POX	U/g	113.60	24.72, 202.48	3
**SOD-CAT-POX**	U/g	114.42	85.66, 143.17	9
MDA	mM/g	1.95	1.53, 2.37	3

CI 95%: 95% confidence interval.

## Data Availability

All data are available within the manuscript and its Supplementary Materials files.

## Supplementary Materials

The following supporting information can be downloaded at: https://www.mdpi.com/article/10.3390/antiox13060692/s1, Supplementary Figure S1: Meta-regression analysis of concentration (as effect size) for DPPH assay for hydroalcoholic extracts; Supplementary Figure S2: Meta-regression analysis of concentration (as effect size) for FRAP, ABTS, ORAC assays for aqueous and hydroalcoholic extracts; Supplementary Figure S3: Meta-regression analysis of concentration (as effect size) for SOD-CAT-POX assays; Supplementary Table S1: Characteristics of 166 individual studies from 8 articles on Phenolic/Flavonoid Compounds’ Profiles in Stevia Extracts.
