# Supplementary material for: Integration of Antioxidant Activity Assays Data of Stevia Leaf Extracts: A Systematic Review and Meta-Analysis
Source: Antioxidants (Basel). 2024 Jun 4;13(6):692. doi: 10.3390/antiox13060692 (PMC11201069; doi:10.3390/antiox13060692)
Supplement: Supplementary file 1 [file antioxidants-13-00692-s001.zip › proofs_SUPPLEMENTARY_Figure_1 (1).pptx]

## Slide 1
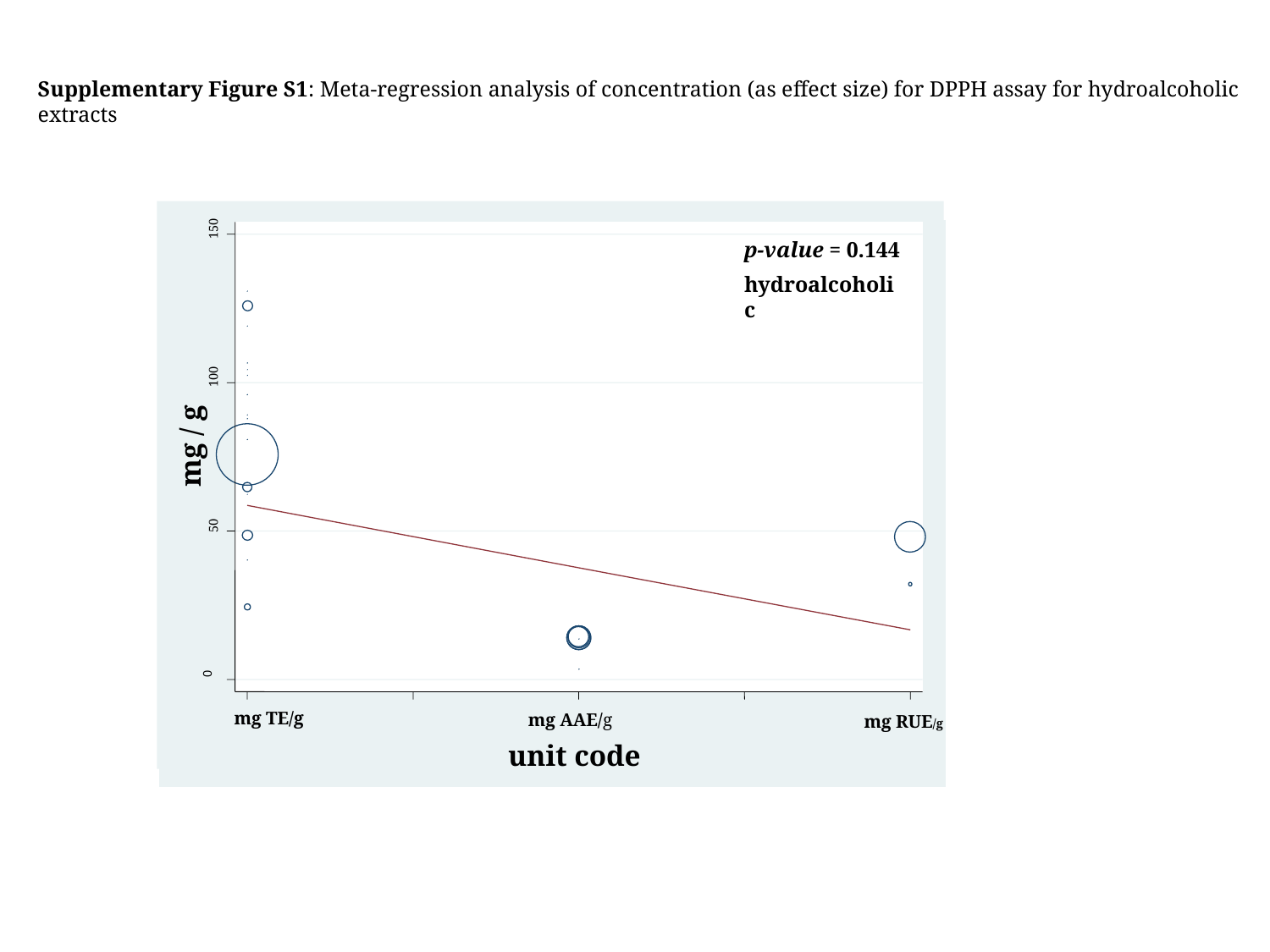

Supplementary Figure S1: Meta-regression analysis of concentration (as effect size) for DPPH assay for hydroalcoholic extracts
150
100
mg / g
50
0
mg TE/g
mg AAE/g
p-value = 0.144
hydroalcoholic
mg RUE/g
unit code
