# Supplementary material for: Integration of Antioxidant Activity Assays Data of Stevia Leaf Extracts: A Systematic Review and Meta-Analysis
Source: Antioxidants (Basel). 2024 Jun 4;13(6):692. doi: 10.3390/antiox13060692 (PMC11201069; doi:10.3390/antiox13060692)
Supplement: Supplementary file 1 [file antioxidants-13-00692-s001.zip › proofs_SUPPLEMENTARY_Figure_2.pptx]

## Slide 1
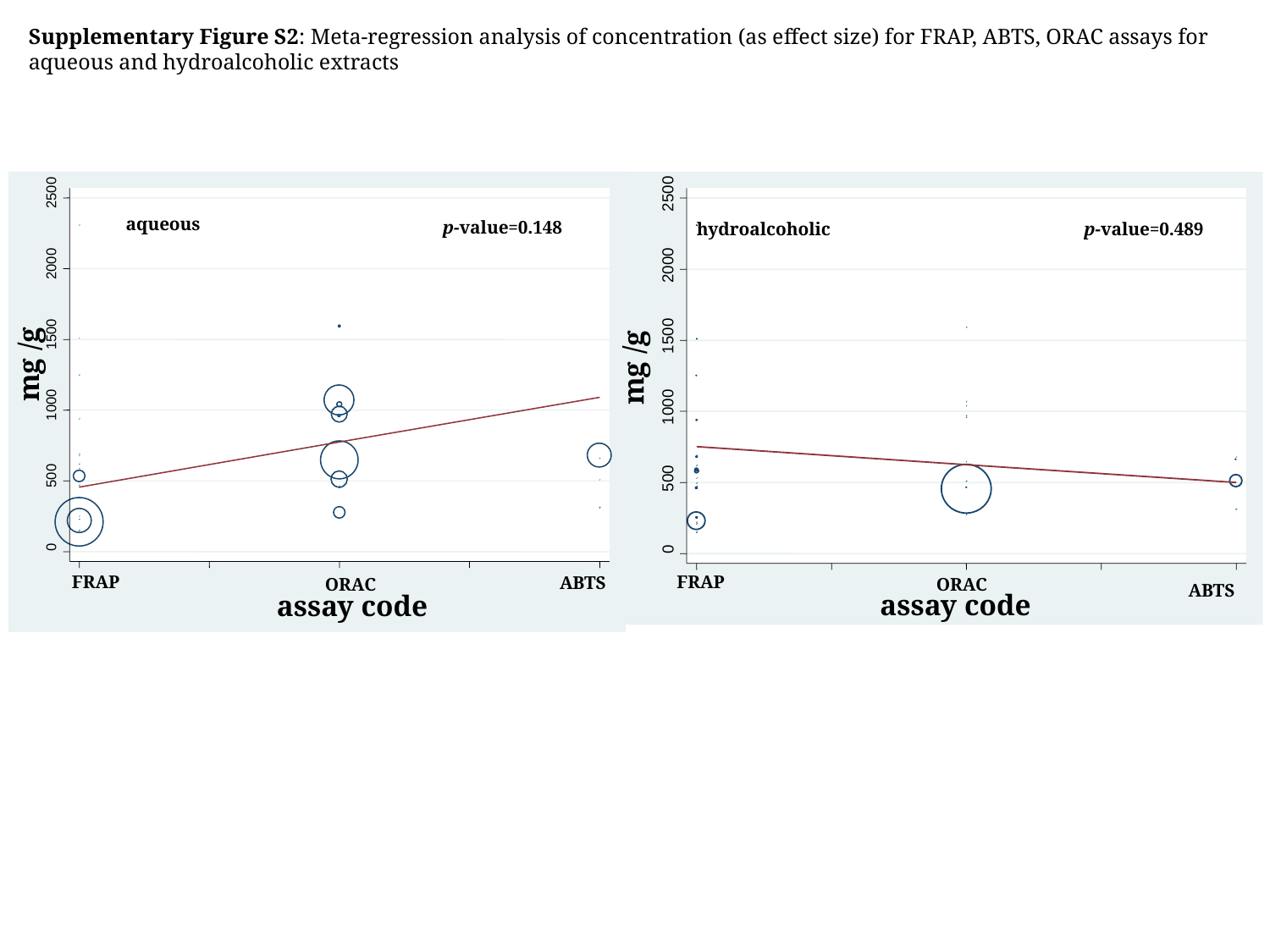

Supplementary Figure S2: Meta-regression analysis of concentration (as effect size) for FRAP, ABTS, ORAC assays for aqueous and hydroalcoholic extracts
2500
2000
1500
1000
500
0
assay code
p-value=0.148
mg /g
FRAP
ABTS
ORAC
aqueous
2500
2000
1500
1000
500
0
hydroalcoholic
p-value=0.489
mg /g
FRAP
ORAC
ABTS
assay code
p-value=0,489
hydroalcoholic
