# Supplementary material for: Integration of Antioxidant Activity Assays Data of Stevia Leaf Extracts: A Systematic Review and Meta-Analysis
Source: Antioxidants (Basel). 2024 Jun 4;13(6):692. doi: 10.3390/antiox13060692 (PMC11201069; doi:10.3390/antiox13060692)
Supplement: Supplementary file 1 [file antioxidants-13-00692-s001.zip › proofs_SUPPLEMENTARY_Figure_3.pptx]

## Slide 1
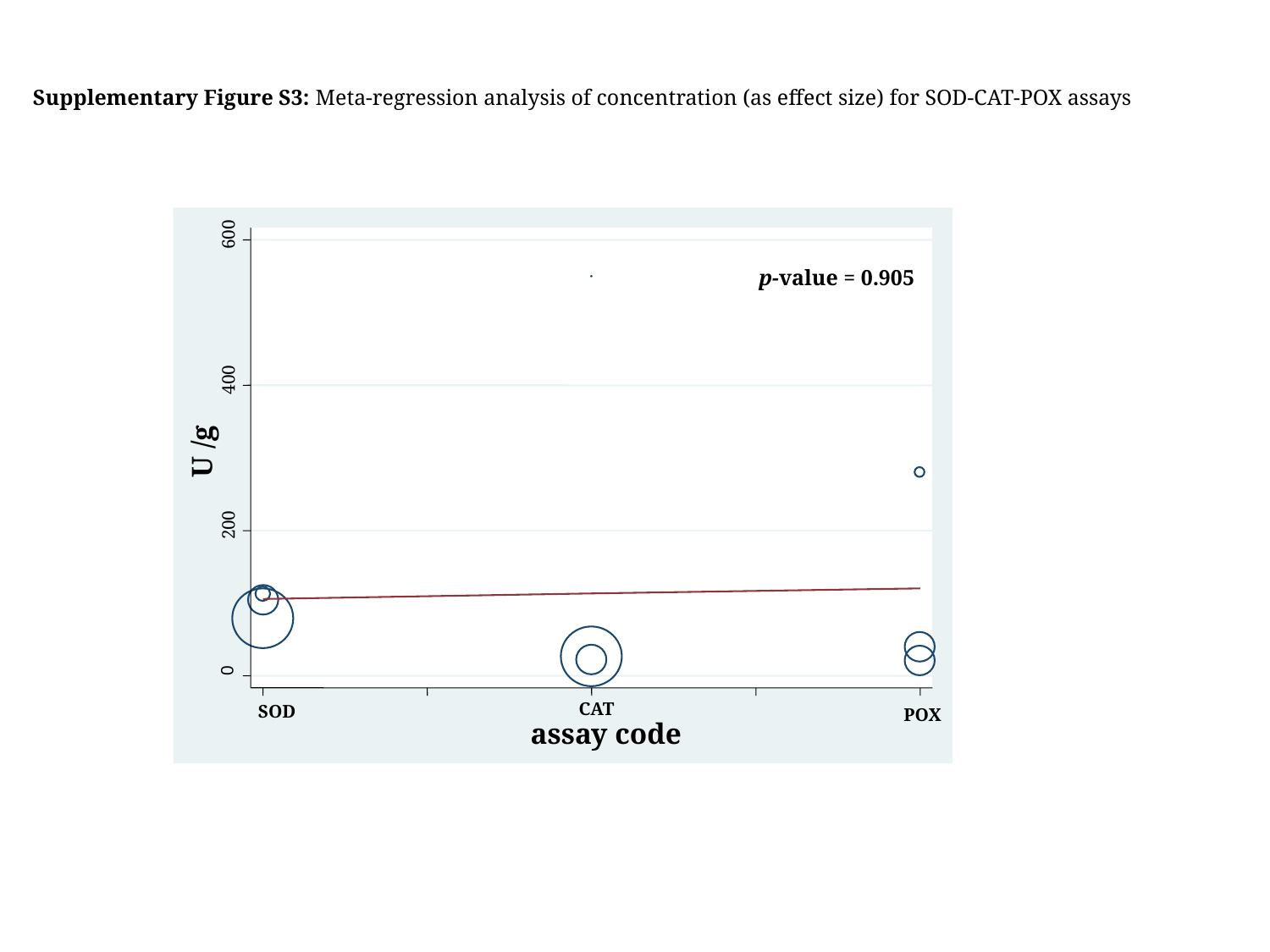

Supplementary Figure S3: Meta-regression analysis of concentration (as effect size) for SOD-CAT-POX assays
600
400
200
0
assay code
U /g
CAT
SOD
POX
p-value = 0.905
