# Supplementary material for: Integration of Antioxidant Activity Assays Data of Stevia Leaf Extracts: A Systematic Review and Meta-Analysis
Source: Antioxidants (Basel). 2024 Jun 4;13(6):692. doi: 10.3390/antiox13060692 (PMC11201069; doi:10.3390/antiox13060692)
Supplement: Supplementary file 1 [file antioxidants-13-00692-s001.zip › proofs_SUPPLEMENTARY_Table 1.pdf]

Supplementary Table S1: Characteristics of 166 individual studies from 8 articles on Phenolic/Flavonoid Compounds' Profiles in Stevia Extracts										
**Author**	**Specific Characteristics of stevia samples**	**publication year**	**Compound**	**Phenol /flavonoid**	**extract**	**Given Concentration (µg/g dry sample)**	**Given Concentration (mg/g dry sample)**	**Given Concentration (µg/mL extract)**	**Given Concentration (mg CGAE/g dry sample)**	**Transformed Concentration mg/g dry**
Barroso et al. [1]	Frozen fresh	2018	3-Caffeoylquinic acid	phenol	hydroalcoholic / 80% methanol		2.28			2.28
Barroso et al. [1]	Dried	2018	3-Caffeoylquinic acid	phenol	hydroalcoholic / 80% methanol		4.57			4.57
Barroso et al. [1]	Powder-leaf	2015	3-Caffeoylquinic acid	phenol	aqueous				0.45	
Barroso et al. [1]	Powder-leaf-organic	2015	3-Caffeoylquinic acid	phenol	aqueous				2.12	
Barroso et al. [1]	Leaf-company1	2015	3-Caffeoylquinic acid	phenol	aqueous				5.43	
Barroso et al. [1]	Leaf-company2	2015	3-Caffeoylquinic acid	phenol	aqueous				4.58	
Barroso et al. [1]	Leaf-Peru	2015	3-Caffeoylquinic acid	phenol	aqueous				1.43	
Gaweł-Bęben et al. [2]		2015	3-Caffeoylquinic acid	phenol	ethanol		0.3			0.3
Barroso et al. [1]	Frozen fresh	2018	3,4-dicaffeoylquinic acid	phenol	hydroalcoholic / 80% methanol		2.01			2.01
Barroso et al. [1]	Dried	2018	3,4-dicaffeoylquinic acid	phenol	hydroalcoholic / 80% methanol		5.27			5.27

Bender et al. [3]	Powder-leaf	2015	3,4-dicaffeoylquinic acid	phenol	aqueous				0.34	
Bender et al. [3]	Powder-leaf-organic	2015	3,4-dicaffeoylquinic acid	phenol	aqueous				2.25	
Bender et al. [3]	Leaf-company1	2015	3,4-dicaffeoylquinic acid	phenol	aqueous				6.18	
Bender et al. [3]	Leaf-company2	2015	3,4-dicaffeoylquinic acid	phenol	aqueous				3.58	
Bender et al. [3]	Leaf-Peru	2015	3,4-dicaffeoylquinic acid	phenol	aqueous				2.15	
El-Hadary et al. [3]		2021	3,4,5-Methoxy-cinnamic	phenol	hydroalcoholic / 80% ethanol		13.3			13.3
Barroso et al. [1]	Frozen fresh	2018	3,5-dicaffeoylquinic acid	phenolic	hydroalcoholic / 80% methanol		28.39			28.39
Barroso et al. [1]	Dried	2018	3,5-dicaffeoylquinic acid	phenolic	hydroalcoholic / 80% methanol		46.66			46.66
Bender et al. [3]	Powder-leaf	2015	3,5-dicaffeoylquinic acid	phenol	aqueous				10.0	
Bender et al. [3]	Powder-leaf-organic	2015	3,5-dicaffeoylquinic acid	phenol	aqueous				42.9	
Bender et al. [3]	Leaf-company1	2015	3,5-dicaffeoylquinic acid	phenol	aqueous				98.0	
Bender et al. [3]	Leaf-company2	2015	3,5-dicaffeoylquinic acid	phenol	aqueous				80.3	
Bender et al. [3]	Leaf-Peru	2015	3,5-dicaffeoylquinic acid	phenol	aqueous				29.9	

Barroso et al. [1]	Frozen fresh	2018	4-Caffeoylquinic acid	phenol	hydroalcoholic / 80% methanol		1.64			1.64
Barroso et al. [1]	Dried	2018	4-Caffeoylquinic acid	phenol	hydroalcoholic / 80% methanol		2.78			2.78
Bender et al. [3]	Powder-leaf	2015	4-Caffeoylquinic acid	phenol	aqueous				4.81	
Bender et al. [3]	Powder-leaf-organic	2015	4-Caffeoylquinic acid	phenol	aqueous				21.22	
Bender et al.[3]	Leaf-company1	2015	4-Caffeoylquinic acid	phenol	aqueous				41.56	
Bender et al.[3]	Leaf-company2	2015	4-Caffeoylquinic acid	phenol	aqueous				36.19	
Bender et al.[3]	Leaf-Peru	2015	4-Caffeoylquinic acid	phenol	aqueous				6.55	
Barroso et al. [1]	Dried	2018	4,5-dicaffeoylquinic acid	phenol	hydroalcoholic / 80% methanol		3.6			3.6
Barroso et al. [1]	Frozen fresh	2018	4,5-dicaffeoylquinic acid	phenol	hydroalcoholic / 80% methanol		8.59			8.59
Bender et al. [3]	Powder-leaf	2015	4,5-dicaffeoylquinic acid	phenol	aqueous				1.51	
Bender et al. [3]	Powder-leaf-organic	2015	4,5-dicaffeoylquinic acid	phenol	aqueous				18.91	
Bender et al. [3]	Leaf-company1	2015	4,5-dicaffeoylquinic acid	phenol	aqueous				40.40	
Bender et al. [3]	Leaf-company2	2015	4,5-dicaffeoylquinic acid	phenol	aqueous				21.65	
Bender et al. [3]	Leaf-Peru	2015	4,5-dicaffeoylquinic acid	phenol	aqueous				18.77	

Karakose et al. [4]	Italy/Aug 11, 2011	2015	4,5-dicaffeoylquinic acid	phenol	organic/ chloroform-methanol		11.55			11.55
Karakose et al. [4]	Greece/2011	2015	4,5-dicaffeoylquinic acid	phenol	organic/ chloroform-methanol		12.34			12.34
Karakose et al. [4]	Spain	2015	4,5-dicaffeoylquinic acid	phenol	organic/ chloroform-methanol		12.75			12.75
Karakose et al. [4]	Italy/July 07, 2011	2015	4,5-dicaffeoylquinic acid	phenol	organic/ chloroform-methanol		12.32			12.32
Karakose et al. [4]	Greece/2010	2015	4,5-dicaffeoylquinic acid	phenol	organic/ chloroform-methanol		16.81			16.81
Barroso et al. [1]	Frozen fresh	2018	5-Caffeoylquinic acid	phenol	hydroalcoholic / 80% methanol		32.64	[4]		32.64
Barroso et al. [1]	Dried	2018	5-Caffeoylquinic acid	phenol	hydroalcoholic / 80% methanol		44.4			44.4
Bender et al. [3]	Powder-leaf	2015	5-Caffeoylquinic acid	phenol	aqueous				0.20	
Bender et al. [3]	Powder-leaf-organic	2015	5-Caffeoylquinic acid	phenol	aqueous				1.77	
Bender et al. [3]	Leaf-company1	2015	5-Caffeoylquinic acid	phenol	aqueous				3.15	
Bender et al. [3]	Leaf-company2	2015	5-Caffeoylquinic acid	phenol	aqueous				1.91	
Bender et al. [3]	Leaf-Peru	2015	5-Caffeoylquinic acid	phenol	aqueous				1.16	
Karakose et al. [4]	Italy/Aug 11, 2011	2015	5-Caffeoylquinic acid	phenol	organic/ chloroform-methanol		26.92			26.92
Karakose et al. [4]	Greece/2011	2015	5-Caffeoylquinic acid	phenol	organic/ chloroform-methanol		24.65			24.65

Karakose et al. [4]	Spain	2015	5-Caffeoylquinic acid	phenol	organic/ chloroform-methanol		20.63			20.63
Karakose et al. [4]	Italy/July 07, 2011	2015	5-Caffeoylquinic acid	phenol	organic/ chloroform-methanol		18.43			18.43
Karakose et al. [4]	Greece/2010	2015	5-Caffeoylquinic acid	phenol	organic/ chloroform-methanol		26.34			26.34
Bender et al. [3]	Powder-leaf	2015	5-Coumaroyl quinic acid	phenol	aqueous				0.05	
Bender et al. [3]	Powder-leaf-organic	2015	5-Coumaroyl quinic acid	phenol	aqueous				0.07	
Bender et al. [3]	Leaf-company1	2015	5-Coumaroyl quinic acid	phenol	aqueous				0.09	
Bender et al. [3]	Leaf-company2	2015	5-Coumaroyl quinic acid	phenol	aqueous				0.08	
Bender et al. [3]	Leaf-Peru	2015	5-Coumaroyl quinic acid	phenol	aqueous				0.06	
El-Hadary et al. [5]		2021	Alpha-coumaric	phenol	hydroalcoholic / 80% ethanol		0.043			0.43
Gaweł-Bęben et al. [2]		2015	Benzoic acid derivatives	phenol	aqueous		0.1			0.1
Gaweł-Bęben et al. [2]		2015	Benzoic acid derivatives	phenol	ethanol		0.05			0.05
Andrade et al. [6]		2021	Caffeic acid	phenol	aqueous	776.9				0.78
Andrade et al.[6]		2021	Caffeic acid	phenol	hydroalcoholic / 12% ethanol	515.9				0.515
Gaweł-Bęben et al. [2]		2021	Caffeic acid	phenol	aqueous		0.29			0.29
Gaweł-Bęben et al. [2]		2021	Caffeic acid	phenol	ethanol		0.06			0.06
Gaweł-Bęben et al. [2]		2021	Caffeic acid	phenol	glycol-aqueous		0.19			0.19

Alshawwa et al. [7]		2022	Caffeic acid	phenol	organic/ chloroform-methanol			0.23		0.001
El-Hadary et al. [5]		2021	Caffeic acid	phenol	hydroalcoholic / 80% ethanol		1.53			1.53
Barroso et al. [1]	Frozen fresh	2018	Caffeic acid derivatives	phenol	hydroalcoholic / 80% methanol		0.17			0.17
Barroso et al. [1]	Dried	2018	Caffeic acid derivatives	phenol	hydroalcoholic / 80% methanol		0.43			0.43
Gaweł-Bęben et al. [2]		2021	Caffeic acid derivatives	phenol	aqueous		0.06			0.06
Gaweł-Bęben et al. [2]		2021	Caffeic acid derivatives	phenol	ethanol		0.03			0.03
Gaweł-Bęben et al. [2]		2021	Caffeic acid derivatives	phenol	glycol-aqueous		0.36			0.36
Bender et al. [3]	Powder-leaf	2015	Caffeoyl shikimic acid	phenol	aqueous				0.11	
Bender et al. [3]	Powder-leaf-organic	2015	Caffeoyl shikimic acid	phenol	aqueous				0.27	
Bender et al. [3]	Leaf-company1	2015	Caffeoyl shikimic acid	phenol	aqueous				0.50	
Bender et al. [3]	Leaf-company2	2015	Caffeoyl shikimic acid	phenol	aqueous				0.76	
Bender et al. [3]	Leaf-Peru	2015	Caffeoyl shikimic acid	phenol	aqueous				0.16	
Barroso et al. [1]	Frozen fresh	2018	caffeoyl-2,7-anhydro-3-deoxy-2-octulopyranosonic acid	phenol	hydroalcoholic / 80% methanol		0.3			0.3
Barroso et al. [1]	Dried	2018	caffeoyl-2,7-anhydro-3-deoxy-2-	phenol	hydroalcoholic / 80% methanol		1.37			1.37

			octulopyranosonic acid							
Gaweł-Bęben et al. [2]		2021	Campherol derivatives	flavonoid	ethanol		0.15			0.15
Gaweł-Bęben et al. [2]		2021	Campherol derivatives	flavonoid	glycol-aqueous		0.23			0.23
Gaweł-Bęben et al. [2]		2021	Catechin	flavonoid	aqueous		0.24			0.24
Alshawwa et al. [7]		2022	Catechin	phenol	organic/ chloroform-methanol			0.73		0.003
El-Hadary et al. [5]		2021	Catechin	flavonoid	hydroalcoholic / 80% ethanol		143.8			143.8
Gaweł-Bęben et al. [2]		2021	Catechin derivatives	flavonoid	aqueous		0.29			0.29
Gaweł-Bęben et al. [2]		2021	Catechin derivatives	flavonoid	ethanol		0.12			0.12
El-Hadary et al. [5]		2021	Catechol	phenol	hydroalcoholic / 80% ethanol		5.9			5.9
Andrade et al. [6]		2021	3-Caffeoylquinic acid	phenol	aqueous	5183.51				5.18
Andrade et al. [6]		2021	3-Caffeoylquinic acid	phenol	hydroalcoholic / 12% ethanol	766.55				0.77
Alshawwa et al. [7]		2022	3-Caffeoylquinic acid	phenol	organic/ chloroform-methanol			0.20		0.001
El-Hadary et al. [5]		2021	3-Caffeoylquinic acid	phenol	hydroalcoholic / 80% ethanol		38.68			38.68
Gaweł-Bęben et al. [2]		2021	Chlorogenic acid derivatives	phenol	ethanol		0.14			0.14

Karakose et al. [8]		2011	Chlorogenic acid derivatives	phenol	organic/ chloroform-methanol	370				
Karakose et al. [4]	Italy/Aug 11, 2011	2015	cis-4,5-dicaffeoylquinic acid	phenol	organic/ chloroform-methanol		0.08			0.08
Karakose et al. [4]	Italy/Aug 11, 2011	2015	cis-4,5-dicaffeoylquinic acid	phenol	organic/ chloroform-methanol		0.15			0.15
Karakose et al. [4]	Greece/2011	2015	cis-4,5-dicaffeoylquinic acid	phenol	organic/ chloroform-methanol		0.08			0.08
Karakose et al. [4]	Greece/2011	2015	cis-4,5-dicaffeoylquinic acid	phenol	organic/ chloroform-methanol		0.17			0.17
Karakose et al. [4]	Spain	2015	cis-4,5-dicaffeoylquinic acid	phenol	organic/ chloroform-methanol		0.29			0.29
Karakose et al. [4]	Spain	2015	cis-4,5-dicaffeoylquinic acid	phenol	organic/ chloroform-methanol		0.58			0.58
Karakose et al. [4]	Italy/July 07, 2011	2015	cis-4,5-dicaffeoylquinic acid	phenol	organic/ chloroform-methanol		0.22			0.22
Karakose et al. [4]	Italy/July 07, 2011	2015	cis-4,5-dicaffeoylquinic acid	phenol	organic/ chloroform-methanol		0.28			0.28
Karakose et al. [4]	Greece/2010	2015	cis-4,5-dicaffeoylquinic acid	phenol	organic/ chloroform-methanol		0.45			0.45
Karakose et al. [4]	Greece/2010	2015	cis-4,5-dicaffeoylquinic acid	phenol	organic/ chloroform-methanol		0.45			0.45
Karakose et al. [4]	Italy/Aug 11, 2011	2015	cis-5-Caffeoylquinic acid	phenol	organic/ chloroform-methanol		1.47			1.47
Karakose et al. [4]	Greece/2011	2015	cis-5-Caffeoylquinic acid	phenol	organic/ chloroform-methanol		1.74			1.74

Karakose et al. [4]	Spain	2015	cis-5-Caffeoylquinic acid	phenol	organic/ chloroform-methanol		1.36			1.36
Karakose et al. [4]	Italy/July 07, 2011	2015	cis-5-Caffeoylquinic acid	phenol	organic/ chloroform-methanol		1.2			1.2
Karakose et al. [4]	Greece/2010	2015	cis-5-Caffeoylquinic acid	phenol	organic/ chloroform-methanol		1.38			1.38
El-Hadary et al. [5]		2021	Ellagic acid	phenol	hydroalcoholic / 80% ethanol		36.03			36.03
Andrade et al. [6]		2021	Epicatechin	flavonoid	hydroalcoholic / 12% ethanol	9.97				0.01
Gaweł-Bęben et al. [2]		2021	Epicatechin	flavonoid	ethanol		0.11			0.11
Andrade et al. [6]		2021	Ferulic acid	phenol	aqueous	144.32				0.14
Andrade et al. [6]		2021	Ferulic acid	phenol	hydroalcoholic / 12% ethanol	32.11				0.03
Alshawwa et al. [7]		2022	Ferulic acid	phenol	organic/ chloroform-methanol			3.59		0.013
El-Hadary et al. [5]		2021	Ferulic acid	phenol	hydroalcoholic / 80% ethanol		4.09			4.09
Gaweł-Bęben et al. [2]		2021	Ferulic acid derivatives	phenol	ethanol		0.86			0.86
Gaweł-Bęben et al. [2]		2021	Ferulic acid derivatives	phenol	glycol-aqueous		5.5			5.5
Alshawwa et al. [7]		2022	Gallic acid	phenol	organic/ chloroform-methanol			13.48		0.05
El-Hadary et al. [5]		2021	Gallic acid	phenol	hydroalcoholic / 80% ethanol		0.95			0.95

El-Hadary et al. [5]		2021	Iso-ferulic acid	phenol	hydroalcoholic / 80% ethanol		13.65			13.65
Andrade et al. [6]		2021	Kaempferol	flavonoid	aqueous	47.51				0.05
Andrade et al. [6]		2021	Kaempferol	flavonoid	hydroalcoholic / 12% ethanol	10.49				0.01
Barroso et al. [1]	Dried	2015	kaempferol-3-O-glucoside	flavonoid	hydroalcoholic / 80% methanol		0.37			0.37
Barroso et al. [1]	Dried	2015	kaempferol-O-deoxyhexoside.	flavonoid	hydroalcoholic / 80% methanol		1.31			1.31
Barroso et al. [1]	Dried	2015	kaempferol-O-pentoside	flavonoid	hydroalcoholic / 80% methanol		0.39			0.39
Barroso et al. [1]	Frozen fresh	2015	kaempferol-O-pentoside	flavonoid	hydroalcoholic / 80% methanol		0.79			0.79
Barroso et al. [1]	Frozen fresh	2015	kaempferol-O-pentoside	flavonoid	hydroalcoholic / 80% methanol		0.44			0.44
Gaweł-Bęben et al. [2]		2021	Luteolin	flavonoid	ethanol		0.03			0.03
Gaweł-Bęben et al. [2]		2021	Luteolin derivatives	flavonoid	ethanol		0.01			0.01
Gaweł-Bęben et al. [2]		2021	Luteolin derivatives	flavonoid	glycol-aqueous		0.86			0.86
Andrade et al.[6]		2021	Naringenin	flavonoid	aqueous	3268.78				3.27
Andrade et al. [6]		2022	Naringenin	flavonoid	hydroalcoholic / 12% ethanol	1805.24				1.81
Andrade et al. [6]		2023	p-Coumaric acid	phenol	aqueous	140.58				0.14

Andrade et al. [6]		2024	p-Coumaric acid	phenol	hydroalcoholic / 12% ethanol	47.9				0.05
Andrade et al. [6]		2025	p-Coumaric acid	phenol	organic/ chloroform-methanol			6.15		0.02
El-Hadary et al. [5]		2021	p-Coumaric acid	phenol	hydroalcoholic / 80% ethanol		1.45			1.45
El-Hadary et al. [5]		2021	P-OH-benzoic	phenol	hydroalcoholic / 80% ethanol		4.97			4.97
Andrade et al. [6]		2021	Protocatechuic acid	phenol	aqueous	3.11				0.003
Andrade et al. [6]		2021	Protocatechuic acid	phenol	hydroalcoholic / 12% ethanol	216.72				0.22
Gaweł-Bęben et al. [2]		2021	Protocatechuic acid	phenol	aqueous		0.12			0.12
Alshawwa et al. [7]		2022	Protocatechuic acid	phenol	organic/ chloroform-methanol			1.25		0.004
El-Hadary et al. [5]		2021	Protocatechuic acid	phenol	hydroalcoholic / 80% ethanol		6.91			6.91
El-Hadary et al. [5]		2021	Pyrogallol	phenol	hydroalcoholic / 80% ethanol		20.23			20.23
Barroso et al. [1]	Frozen fresh	2015	quercetin-3-O-glucoside	flavonoid	hydroalcoholic / 80% methanol		1.62			1.62
Barroso et al. [1]	Dried	2015	quercetin-3-O-glucoside	flavonoid	hydroalcoholic / 80% methanol		2.92			2.92
Barroso et al. [1]	Frozen fresh	2015	quercetin-3-O-rhamnoside	flavonoid	hydroalcoholic / 80% methanol		5.38			5.38
Barroso et al. [1]	Frozen fresh	2015	quercetin-3-O-xyloside	flavonoid	hydroalcoholic / 80% methanol		1.96			1.96

Barroso et al. [1]	Dried	2015	quercetin-3-O-xyloside	flavonoid	hydroalcoholic / 80% methanol		2.47			2.47
Barroso et al. [1]	Frozen fresh	2015	quercetin-O-pentosyl-deoxyhexoside	flavonoid	hydroalcoholic / 80% methanol		0.76			0.76
Gaweł-Bęben et al. [2]		2021	Rozmaric acid	phenol	ethanol		0.36			0.36
Gaweł-Bęben et al. [2]		2021	Rozmaric acid derivatives	phenol	ethanol		0.42			0.42
Gaweł-Bęben et al. [2]		2021	Rozmaric acid derivatives	phenol	glycol-aqueous		4.95			4.95
Andrade et al. [6]		2021	Rutin	flavonoid	hydroalcoholic / 12% ethanol	2.31				0.23
Gaweł-Bęben et al. [2]		2021	Rutin	flavonoid	glycol-aqueous		0.17			0.17
Gaweł-Bęben et al. [2]		2021	Rutin derivatives	flavonoid	ethanol		0.12			0.12
Gaweł-Bęben et al. [2]		2021	Rutin derivatives	flavonoid	glycol-aqueous		1.05			1.05
El-Hadary et al. [5]		2021	Salicylic acid	phenol	hydroalcoholic / 80% ethanol		28.79			28.79
Gaweł-Bęben et al. [2]		2021	Salicylic acid derivatives	phenol	aqueous		0.06			0.06
Alshawwa et al. [7]		2022	Syringic acid	phenol	organic/ chloroform-methanol			7.83		7.83
Andrade et al. [6]		2021	Vanillic acid	phenol	aqueous	247.87				0.247
Andrade et al. [6]		2021	Vanillic acid	phenol	hydroalcoholic / 12% ethanol	46.37				0.05
El-Hadary et al. [5]		2021	Vanillic acid	phenol	hydroalcoholic / 80% ethanol		1.83			1.83

Andrade et al.[6]		2021	Vanillin	phenol	aqueous	448.47				0.45
Andrade et al. [6]		2021	Vanillin	phenol	hydroalcoholic / 12% ethanol	88.68				0.09
CGAE: Chlorogenic Acid Equivalents; hydroalcoholic: solvent mixture of water and alcohol										

References

1. Barroso, M.R.; Martins, N.; Barros, L.; Antonio, A.L.; Rodrigues, M.Â.; Sousa, M.J.; Santos-Buelga, C.; Ferreira, I.C.F.R. Assessment of the Nitrogen Fertilization Effect on Bioactive Compounds of Frozen Fresh and Dried Samples of Stevia Rebaudiana Bertoni. *Food Chem* **2018**, *243*, 208–213, doi:10.1016/j.foodchem.2017.09.137.
2. Gaweł-Bęben, K.; Bujak, T.; Nizioł-Łukaszewska, Z.; Antosiewicz, B.; Jakubczyk, A.; Karaś, M.; Rybczyńska, K. Stevia Rebaudiana Bert. Leaf Extracts as a Multifunctional Source of Natural Antioxidants. *Molecules* **2015**, *20*, 5468–5486, doi:10.3390/molecules20045468.
3. Bender, C.; Graziano, S.; Zimmermann, B.F. Study of Stevia Rebaudiana Bertoni Antioxidant Activities and Cellular Properties. *Int J Food Sci Nutr* **2015**, *66*, 553–558, doi:10.3109/09637486.2015.1038223.
4. Karaköse, H.; Jaiswal, R.; Deshpande, S.; Kuhnert, N. Investigation of the Photochemical Changes of Chlorogenic Acids Induced by Ultraviolet Light in Model Systems and in Agricultural Practice with Stevia Rebaudiana Cultivation as an Example. *J Agric Food Chem* **2015**, *63*, 3338–3347, doi:10.1021/acs.jafc.5b00838.
5. El-Hadary, A.; Sitohy, M. Safely Effective Hypoglycemic Action of Stevia and Turmeric Extracts on Diabetic Albino Rats. *J Food Biochem* **2021**, *45*, doi:10.1111/jfbc.13549.
6. Andrade, J.K.S.; Barros, R.G.C.; Rezende, Y.R.R.S.; Nogueira, J.P.; de Oliveira, C.S.; Gualberto, N.C.; Narain, N. Evaluation of Bioactive Compounds, Phytochemicals Profile and Antioxidant Potential of the Aqueous and Ethanolic Extracts of Some Traditional Fruit Tree Leaves Used in Brazilian Folk Medicine. *Food Research International* **2021**, *143*, doi:10.1016/j.foodres.2021.110282.

7. Alshawwa, S.Z.; Mohammed, E.J.; Hashim, N.; Sharaf, M.; Selim, S.; Alhuthali, H.M.; Alzahrani, H.A.; Mekky, A.E.; Elharrif, M.G. In Situ Biosynthesis of Reduced Alpha Hematite (α-Fe2O3) Nanoparticles by Stevia Rebaudiana L. Leaf Extract: Insights into Antioxidant, Antimicrobial, and Anticancer Properties. *Antibiotics (Basel)* **2022**, *11*, doi:10.3390/ANTIBIOTICS11091252.
8. Karaköse, H.; Jaiswal, R.; Kuhnert, N. Characterization and Quantification of Hydroxycinnamate Derivatives in Stevia Rebaudiana Leaves by LC-MS n. *J Agric Food Chem* **2011**, *59*, 10143–10150, doi:10.1021/jf202185m.
